# The bHLH transcription factor SPATULA enables cytokinin signaling, and both activate auxin biosynthesis and transport genes at the medial domain of the gynoecium

**DOI:** 10.1371/journal.pgen.1006726

**Published:** 2017-04-07

**Authors:** J. Irepan Reyes-Olalde, Víctor M. Zúñiga-Mayo, Joanna Serwatowska, Ricardo A. Chavez Montes, Paulina Lozano-Sotomayor, Humberto Herrera-Ubaldo, Karla L. Gonzalez-Aguilera, Patricia Ballester, Juan José Ripoll, Ignacio Ezquer, Dario Paolo, Alexander Heyl, Lucia Colombo, Martin F. Yanofsky, Cristina Ferrandiz, Nayelli Marsch-Martínez, Stefan de Folter

**Affiliations:** 1 Unidad de Genómica Avanzada (LANGEBIO), Centro de Investigación y de Estudios Avanzados del Instituto Politécnico Nacional (CINVESTAV-IPN), Irapuato, Guanajuato, México; 2 Instituto de Biología Molecular y Celular de Plantas, CSIC-UPV Universidad Politécnica de Valencia, Valencia, Spain; 3 Division of Biological Sciences, University of California San Diego, La Jolla, California, United States of America; 4 Dipartimento di Bioscienze, Università degli Studi di Milano, Milan, Italy; 5 Biology Department, Adelphi University, Garden City, New York, United States of America; 6 Departamento de Biotecnología y Bioquímica, CINVESTAV-IPN, Irapuato, Guanajuato, México; The University of North Carolina at Chapel Hill, UNITED STATES

## Abstract

Fruits and seeds are the major food source on earth. Both derive from the gynoecium and, therefore, it is crucial to understand the mechanisms that guide the development of this organ of angiosperm species. In *Arabidopsis*, the gynoecium is composed of two congenitally fused carpels, where two domains: medial and lateral, can be distinguished. The medial domain includes the carpel margin meristem (CMM) that is key for the production of the internal tissues involved in fertilization, such as septum, ovules, and transmitting tract. Interestingly, the medial domain shows a high cytokinin signaling output, in contrast to the lateral domain, where it is hardly detected. While it is known that cytokinin provides meristematic properties, understanding on the mechanisms that underlie the cytokinin signaling pattern in the young gynoecium is lacking. Moreover, in other tissues, the cytokinin pathway is often connected to the auxin pathway, but we also lack knowledge about these connections in the young gynoecium. Our results reveal that cytokinin signaling, that can provide meristematic properties required for CMM activity and growth, is enabled by the transcription factor SPATULA (SPT) in the medial domain. Meanwhile, cytokinin signaling is confined to the medial domain by the cytokinin response repressor ARABIDOPSIS HISTIDINE PHOSPHOTRANSFERASE 6 (AHP6), and perhaps by ARR16 (a type-A ARR) as well, both present in the lateral domains (presumptive valves) of the developing gynoecia. Moreover, SPT and cytokinin, probably together, promote the expression of the auxin biosynthetic gene *TRYPTOPHAN AMINOTRANSFERASE OF ARABIDOPSIS 1* (*TAA1*) and the gene encoding the auxin efflux transporter PIN-FORMED 3 (PIN3), likely creating auxin drainage important for gynoecium growth. This study provides novel insights in the spatiotemporal determination of the cytokinin signaling pattern and its connection to the auxin pathway in the young gynoecium.

## Author summary

Most of our food comes from fruits and seeds, derived from a fertilized gynoecium. Therefore, understanding the mechanisms that control gynoecium development is of crucial importance. The Arabidopsis gynoecium has two fused carpels, with a medial domain between them, and a lateral domain consisting of the carpel walls. All the tissues that are involved in reproduction arise from the carpel margin meristem in the medial domain. The phytohormone cytokinin provides meristematic activity to cells, and interestingly, in a young gynoecium, the medial, but not the lateral, domain presents strong cytokinin signaling. One question that comes to mind is how this pattern is defined. This work demonstrates that the transcription factor SPATULA enables cytokinin signaling at the medial domain, while cytokinin signaling repressors are present in the lateral domain. A second question is whether and how cytokinin in the medial domain communicates with auxin, an important phytohormone for tissue differentiation. We found that cytokinin and SPT activate auxin biosynthesis and transport genes. The integration of these findings gives the first gene regulatory network acting during early gynoecium development. This network is most likely conserved in flowering plants, and can provide insights of molecular processes that are key for food production.

## Introduction

Angiosperms (flowering plants) are the most successful group of land plants on earth. In these species, flowers are formed, which normally produce a pistil or gynoecium, the female reproductive part of the flower, in their inner floral whorl. The gynoecium is responsible for fruit production and the formation, protection and dispersal of the seeds. Fruit and seeds are a major food source. Therefore, understanding the mechanisms that control gynoecium development in angiosperm species is of crucial importance.

**Fig 1:**
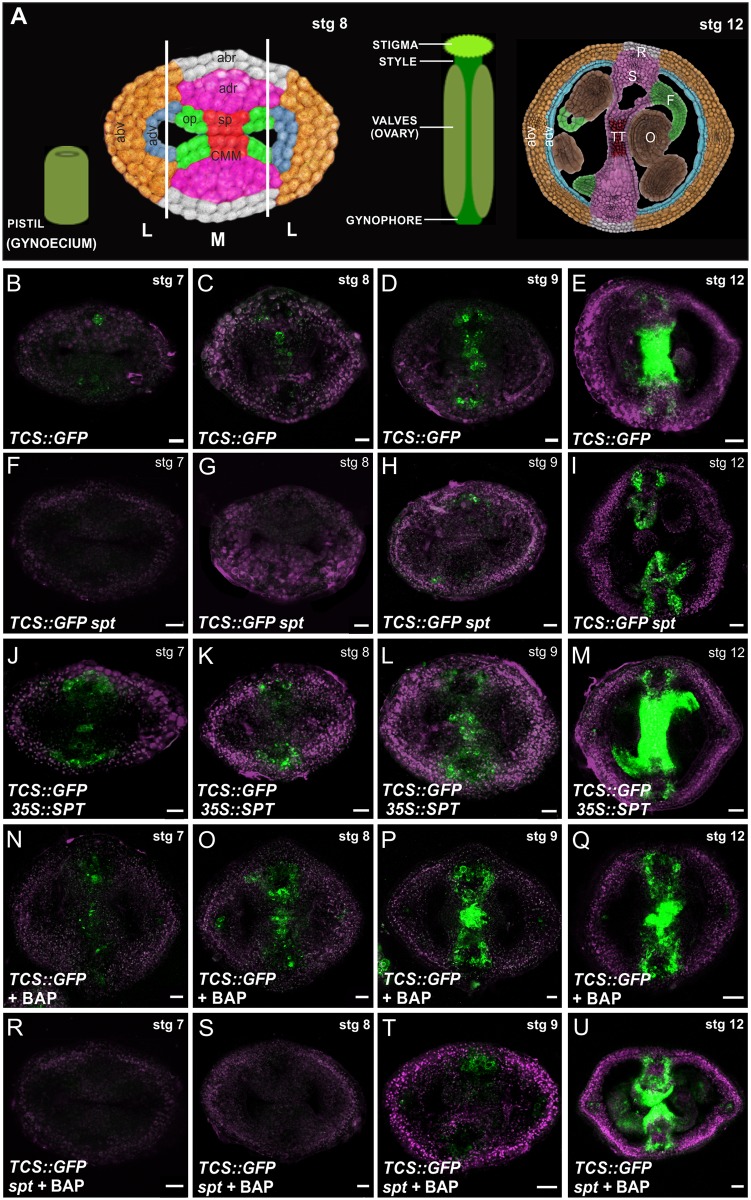
Overview of the gynoecium and SPT is necessary for cytokinin signaling in the young gynoecium. **(****A)** Schematic overview and false-coloured transverse section of a stage 8 and of a stage 12 *Arabidopsis thaliana* gynoecium (pistil). The medial (M) and lateral (L) domains of the gynoecium are indicated. The CMM in the medial domain (stage 8 gynoecium; left side) is indicated and its derived structures can be seen in a stage 12 gynoecium (right side). L, lateral domain; M, medial domain. Orange, abaxial valve (abv); blue, adaxial valve (adv); white, abaxial replum (abr); pink, adaxial replum (adr); green, ovule primordium (op); red, septum primordium (sp); CMM, carpel margin meristem; septum (S); replum (R); transmitting tract (TT); ovule (O); funiculus (F). **(B-M)** Expression of the cytokinin response reporter *TCS*::*GFP* in transverse sections of gynoecia at stage 7, 8, 9, and 12 of wild-type **(B-E)**, *spt-2* **(F-I)**, and *35S*::*SPT* **(J-M)**.**(N-U)** Expression of the reporter *TCS*::*GFP* in transverse sections of gynoecia at stage 7, 8, 9, and 12, after 48 hours of 6-benzylaminopurine (BAP; a synthetic cytokinin) treatment in wild-type **(N-Q)** and *spt-2* **(R-U)**. Scale bars: 20 μm (E, I, M, Q, U), 10 μm (B-D, F-H, J-L, N-P, R-T).

In *Arabidopsis*, the gynoecium is composed of two congenitally fused carpels and from top to bottom we identify the stigma and style, the ovary and the gynophore (in the apical-basal axis; Fig 1A). A tissue with meristematic properties forms along the fused carpel margins (the so-called medial domain), which is called the carpel margin meristem (CMM). The lateral region of the carpel will eventually develop into valves. The CMM gives rise to all medial tissues, including the replum, placenta, ovules, septum and transmitting tract (Fig 1A) [1–3]. All these tissues are crucial for the reproductive success of the plant; however, our knowledge on the early events controlling CMM activity and medial tissue formation is fragmentary [1,4–6].

We have previously shown that the CMM shows a high transcriptional response to the phytohormone cytokinin [7], a plant hormone that has been shown to promote cell division and maintain an undifferentiated cell state in aerial meristematic tissues [8,9]. Consistent with this, reduced cytokinin levels diminish gynoecium cell proliferation, whereas elevated cytokinin levels promote the proliferation of the medial tissues of the gynoecium [7]. Furthermore, mutations in the cytokinin catabolic genes *CYTOKININ OXIDASE/DEHYDROGENASE* (*CKX*), result in larger floral organ size and increased seed yield owing to an increase in meristem size and ovule-forming placenta activity, respectively [10,11].

The cytokinin signal is perceived and transduced by a multi-step two-component signaling pathway, where the binding of the hormone causes the autophosphorylation of the membrane-bound cytokinin receptors ARABIDOPSIS HISTIDINE KINASES [AHK2, AHK3 and AHK4 (aka CRE1)], followed by a phosphorelay cascade [12–14]. The phosphoryl group gets relayed from the receptors to the ARABIDOPSIS HISTIDINE PHOSPHOTRANSFERASE proteins (AHP1-AHP5), with AHP6 competing for the phosphotransfer (i.e., interfering with cytokinin signaling). The AHP1-AHP5 proteins, which shuttle between the cytosol and the nucleus, phosphorylate the ARABIDOPSIS RESPONSE REGULATOR (ARR) proteins in the nucleus. Phosphorylated type-B ARR proteins work as transcription factors activating cytokinin-responsive genes, including the type-A *ARR* genes, which form a feedback loop negatively regulating cytokinin signaling responses [12–14].

The importance of cytokinin is clear in the shoot apical meristem (SAM), where the gene encoding for the homeodomain transcription factor *SHOOT MERISTEMLESS* (*STM*) is expressed [15]. *STM* is required for SAM initiation and maintenance, in part by activating cytokinin biosynthesis *ISOPENTENIL TRANSFERASE* (*IPT*) genes [16–18]. The cytokinin produced is important for the formation and maintenance of stem cell niches [19–21]. Lack of *STM* results in SAM abortion whereas increased expression enlarges the meristem producing more organs [18,22,23], which also occurs when cytokinin signaling decreases or increases, respectively [18,24].

In the young gynoecium, while a high cytokinin signaling output is detected at the medial domain of the ovary, this output is hardly detected at the lateral domain [7]. However, our understanding about the molecular components that contribute to this pattern of cytokinin signaling in specific regions in the young ovary is far from complete.

Previous studies have shown the key role that auxin plays during gynoecium and fruit development (reviewed in: [5,6,25–27]). Altered or impaired auxin signaling responses lead to dramatic gynoecia and fruit apical-basal and medio-lateral patterning defects, incomplete gynoecial apical fusion, altered style and stigma, apical-basal axis gynoecial patterning defects, the block of fruit growth or pod shattering alterations [28–39].

Recently, auxin and cytokinin have been referred as the `yin and yang`of plant development [13], as they are often regarded as having opposite functions, but act synergistically together producing an output that is more than the sum of each of their independent actions. This is evidenced in meristem development [9,40], root vasculature development [41,42], and *in vitro* organogenesis [43,44], among others. In this scenario, it is thus expected that cytokinin-auxin interplay actively participates in early gynoecium development [6]. However, we lack knowledge on whether and how the cytokinin signaling pathway is integrated with the auxin pathway in the young ovary.

In this work, we investigated molecular elements that contribute to the pattern of cytokinin signaling regions in the young ovary, and the connection of the cytokinin signal to the auxin pathway at the medial domain. Our results support that the competence for cytokinin response in the medial tissue is provided by the bHLH transcription factor SPATULA (SPT), known to be important for early gynoecium development [45–47]. On the other hand, the negative cytokinin signaling regulators AHP6 and ARR16 are expressed at the lateral domain, where cytokinin signaling is barely detected. Furthermore, both cytokinin and SPT activate *TAA1* (an auxin biosynthesis enzyme) and *PIN3* (an auxin transporter).

## Results

### SPATULA is required for cytokinin signaling output at the medial domain

We previously observed expression of the cytokinin signaling reporter *TCS*::*GFP* in the medial tissues, such as CMM, septa primordia, septum, transmitting tract [7], and in cells where the provasculature will arise (Fig 1), but could hardly detect expression in the lateral domain of young gynoecia. Our first question was what determined this spatial pattern of cytokinin signaling. To identify possible regulators of cytokinin signaling in gynoecia, we sought for patterning genes important for early gynoecium development and whose expression pattern overlapped with that of *TCS*::*GFP*.

Strikingly, we found that the expression pattern of the regulatory gene *SPATULA* (*SPT*) largely mirrored that of *TCS*::*GFP* (Fig 1B–1E; S1 Fig) [7,47,48]. *SPT* encodes a bHLH transcription factor, whose function is key in early gynoecium morphogenesis as it participates in CMM, septum and the transmitting tract development [45–47]. *SPT* is expressed since early stages in the CMM and its derived structures [47,48]. The single *spt* mutant shows a reduced number of cells in the CMM, absence of the septum and of the transmitting tract, retarded growth of the gynoecial tube and of vasculature, reduced number of ovules, and apical carpel fusion defects, which finally results in poor seed production [45–47]. In this context, we decided to investigate whether *SPT* was participating in the cytokinin signaling pathway during early gynoecium development.

To do this we analyzed the activity of the *TCS*::*GFP* transgene in *spt* mutant gynoecia (Fig 1F–1I). We used confocal laser scanning microscopy to observe fluorescence signal in transversely hand-sectioned gynoecia at stages 7, 8, 9, and 12; stages according to [49]. Remarkably, during early gynoecium development (stage 7–9), no fluorescence signal was detected in the CMM or septa primordia of *spt* mutants (Fig 1F–1H). On the other hand, TCS activity was increased when *SPT* was constitutively expressed (Fig 1J–1M). Interestingly, whereas the fluorescence signal from the *TCS*::*GFP* reporter was increased upon 48 hrs of exogenous cytokinin treatment (Fig 1N–1Q), no GFP signal increase was observed in cytokinin treated *spt* mutant gynoecia (Fig 1R–1U). Note that in the mature *spt* gynoecia (stage 12), *TCS*::*GFP* fluorescence can be observed at the edges of the defective septa, strongly suggesting that this later signal is non-*SPT* dependent (Fig 1I).

In summary, these results support a positive role for *SPT* in the cytokinin signaling pathway at the CMM and septa primordia during early gynoecium formation.

**Fig 2:**
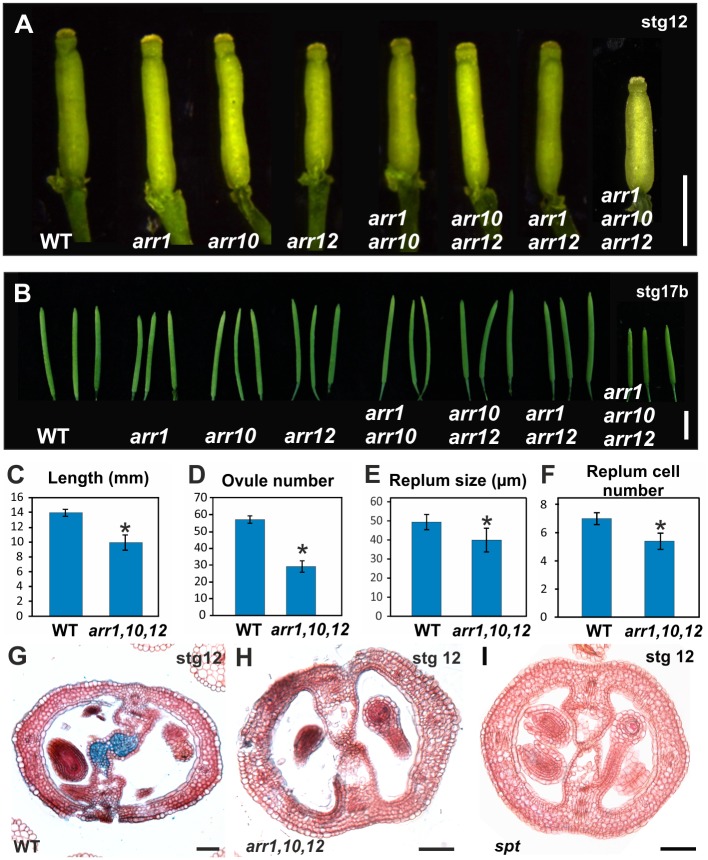
Phenotypes of the type-B *arr* mutants and of the *spt* mutant. **(A)** Mature gynoecium size of wild-type, *arr1*, *arr10*, *arr12*, *arr1 arr10*, *arr10 arr12*, *arr1 arr12*, and *arr1 arr10 arr12*. **(B)** Mature fruit size of wild-type, *arr1*, *arr10*, *arr12*, *arr1 arr10*, *arr10 arr12*, *arr1 arr12*, and *arr1 arr10 arr12*. **(C-F)** Phenotypes of the type-B *arr1 arr10 arr12* triple mutant compared to wild-type (WT): fruit length **(C)**, ovule number **(D)**, replum width **(E)**, and replum cell number **(F)**. **(G-I)** Transverse sections of stage 12 gynoecia of wild-type **(G)**, *arr1 arr10 arr12* (with transmitting tract and septum fusion defects) **(H)**, and *spt-2* **(I)**. Scale bars: 1 mm (A), 5 mm (B), 50 μm (G-I). Error bars represent SD. *P < 0.05 (Student-t test). Sample numbers: (C, D) WT, n = 14 and *arr1 arr10 arr12*, n = 19; (E, F) WT, n = 20 and *arr1 arr10 arr12*, n = 19.

**Fig 3:**
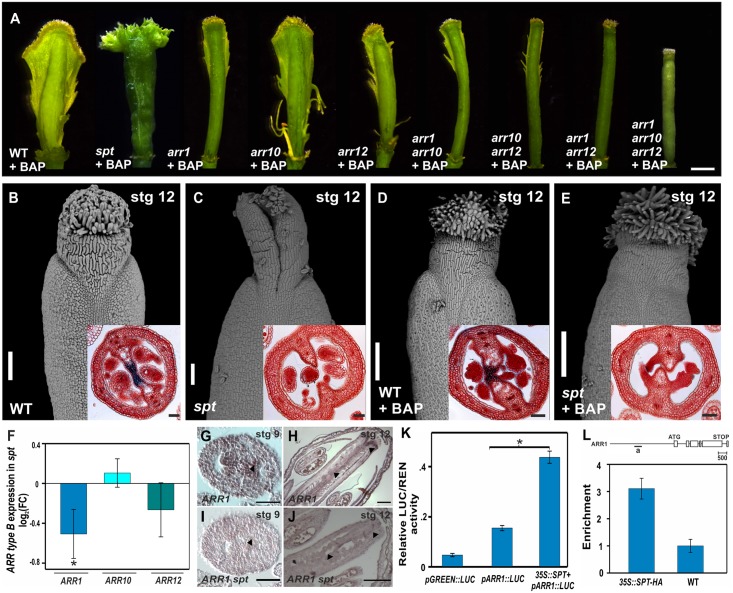
SPT enables cytokinin responses during early gynoecium development and regulates type-B *ARR* gene expression. **(A)** Phenotypes of wild-type, *arr1*, *arr10*, *arr12*, *arr1 arr10*, *arr10 arr12*, *arr1 arr12*, *arr1 arr10 arr12*, and *spt-2* gynoecia three to four weeks after receiving BAP treatment for five to ten days. (**B-E)** Scanning electron microscopy image of wild-type and *spt-2* stage 12 gynoecia one day after either receiving mock **(B, C)** or BAP treatment for only 48 hours **(D, E)**. Insets show a transverse section of the ovary. (**F**) Expression analysis by qRT-PCR of *ARR1*, *ARR10*, and *ARR12* in wild-type and *spt-12* dissected gynoecia. (**G-J**) *In situ* hybridization of type-B *ARR1* mRNA in wild-type **(G, H)** and *spt-2* **(I, J)** floral buds at stages 9 and 12. Arrowheads indicate the detected expression in wild-type and the absence in *spt-2*. **(K**) Luciferase reporter assay in *N*. *benthamiana* leaves co-transformed with *35S*::*SPT* and *pARR1*::*LUC*. Ratio of firefly luciferase (LUC) to Renilla luciferase (REN) activity. **(L)** ChIP experiments against the *ARR1* promoter region (indicated by “a” in the scheme above) using a *35S*::*SPT-HA* line and wild-type. *ACT2/7* served as a negative control. For the LUC assays and qRT-PCR experiments error bars represent the SD based on three biological replicates. ChIP results of one representative experiment are shown; error bars represent the SD of the technical replicates. *P < 0.05 (LUC: Student-t test; qRT-PCR and qPCR: ANOVA). Scale bars: 500 μm (A), 100 μm (B-E, H, J), 50 μm (insets in B-E, G, I).

The cytokinin signaling pathway is necessary for proper gynoecium development Taking into consideration that the lack of *SPT* function causes severe gynoecial developmental defects [45–47] (Figs 2I and 3C) and that, based on our results, it influences cytokinin signaling output (Fig 1), we expected to observe gynoecium morphological alterations when genes in the cytokinin signaling pathway are mutated.

The reporter line *TCS*::*GFP* has a synthetic promoter containing type-B ARR binding sites [50], suggesting that type-B ARRs could be involved in CMM and septum development. We thus analyzed plants with impaired type-B *ARR* function [13]. Out of the 11 type-B ARR transcription factors present in *Arabidopsis*, ARR1, ARR10, and ARR12 are considered to have the main roles, based on cytokinin response assays, studies on root meristem development, and the severe reduction in cytokinin signaling [51–54]. Unfortunately, largely due to gene redundancy, single or double loss-of-function mutants in type-B *ARRs* do not show obvious phenotypic alterations [52]. Indeed, we observed no obvious phenotypic differences between wild type plants and the single loss-of-function mutants *arr1*, *arr10*, and *arr12*, nor with the double loss-of-function mutants *arr1 arr10*, *arr1 arr12*, and *arr10 arr12* (Fig 2 and S2 Fig). In contrast, the type-B *arr1 arr10 arr12* triple mutant plants are severely affected (Fig 2 and S2 Fig). General plant growth is strongly reduced and flower and fruit production is drastically reduced, suggesting that the meristematic activity is affected in this triple mutant (S2 Fig).

We then morphologically characterized the produced gynoecia in the type-B *arr* triple mutant plants. The *arr1 arr10 arr12* background exhibited reproductive defects such as reduced gynoecium and fruit length, reduced replum width, and fewer ovules (Fig 2A–2F), phenotypes not observed in single and double *arr* mutants (Fig 2A and 2B; S2 Fig). Furthermore, thin sections of *arr1 arr10 arr12* triple mutant gynoecia showed septum fusion defects and a reduction of transmitting tract tissue in some gynoecia (Fig 2H), phenotypes that we did not observe in thin sections of gynoecia of single or double *arr* mutants (S3 Fig), confirming the high level of redundancy among type-B ARR transcription factors.

In summary, the analyzed phenotypes provided further evidence supporting the relevance of cytokinin signaling in gynoecium development, and, since some aspects of the triple mutant were reminiscent of the *spt* single mutant, also support the connection between SPT and the cytokinin signaling pathway during gynoecium development.

### SPT and type-B ARRs are required for the cytokinin induced proliferation

We next investigated the functional relevance and nature of the relationship between SPT and cytokinin signaling in the gynoecium using a pharmacological assay to evaluate the cytokinin response competence of the gynoecium. The repeated application of cytokinin to wild-type *Arabidopsis* inflorescences results in tissue overproliferation, causing ectopic outgrowths from the medial domain of the gynoecium; observed three to four weeks after the treatment [7] (Fig 3A). However, this response was affected in the type-B *arr* mutants and in *spt* (Fig 3A).

Single *arr1* and *arr12* mutants presented a very reduced response to the exogenous cytokinin treatments, while *arr10* presented only a mild reduction (Fig 3A). Some proliferation was observed in the double *arr1 arr10*, which resembled the single *arr1* mutant. Some proliferation was also observed in the double *arr10 arr12* mutant, which was a little less than in the single *arr12* mutant. However, no ectopic tissues were produced in the double *arr1 arr12* mutant nor the triple *arr1 arr10 arr12* mutant (Fig 3A). Interestingly, 14 out of 16 *spt* gynoecia (87.5%) also did not show a cytokinin response in the medial domain (Fig 3A), and only a minor proliferation effect was detected in the other two (12.5%) *spt* gynoecia examined (S4B Fig). It is worth noting that, as recently observed by others [55], the cytokinin response of the style and stigma of wild-type and *spt* gynoecia was different to that of the internal ovary. In summary, based on the pharmacological assay, type-B ARR redundancy is observed, with ARR1 and ARR12 playing the major role in the cytokinin response competence of the gynoecium. Moreover, SPT is also a major player in this response. Interestingly, during normal gynoecium development, i.e., no exogenous cytokinin application, morphological defects only become visible when three type-B *ARR* genes are not functional anymore (Fig 3; S2 and S3 Figs), demonstrating that the developmental program active during early gynoecium development is robust. However, the pharmacological assay indicates that the full competence of the gynoecium to respond to the artificial high level of cytokinin needs all type-B ARR proteins to be active, because a decreased response is already visible by removing one type-B ARR (Fig 3A).

We also asked whether an exogenous cytokinin treatment could rescue the developmental defects observed in the *spt* mutant. This mutant has two clear fusion defects: at the apex of the gynoecium and in the internal ovary region [45–47] (Fig 3C). Cytokinin was applied to inflorescences during a 48-hour period only. We observed a virtually complete rescue of the apical closure defect in 20 out of 26 *spt* gynoecia (76.9%), 24 hours after this treatment (Fig 3B–3E; S4C–S4K Fig). However, the *spt* septum defects were not rescued (Fig 3E; S4K Fig). This suggests that the internal *spt* fusion defects in the ovary were most likely not due to reduced cytokinin biosynthesis, and that SPT could be acting at a different level of the cytokinin pathway.

In conclusion, the data together clearly indicate that SPT is necessary for positive cytokinin signaling output (both visualized by the TCS reporter, and as the proliferation response to exogenous cytokinin treatments) in the young gynoecium.

### SPT regulates type-B *ARR* expression

Given that the internal fusion defects of *spt* were not rescued by exogenous cytokinin, together with the resemblances between the *spt* and the type-B *arr1 arr10 arr12* triple mutant phenotypes, and the alteration of cytokinin signaling in the *spt* mutant, we hypothesized whether one of the ways in which SPT could be connected to the cytokinin pathway could be through the regulation of the type-B *ARR* genes.

To test this possibility, we assayed the transcript levels of *ARR1*, *ARR10* and *ARR12* using quantitative real-time reverse transcriptase-mediated polymerase chain reaction (qRT-PCR) from dissected wild-type and *spt* gynoecia, respectively. Whereas this experiment did not reveal clear changes in the expression level of *ARR10*, transcript abundance for *ARR1* and *ARR12* was reduced in *spt* when compared to wild-type (Fig 3F), and both showed a higher relative expression than *ARR10* in wild type gynoecia (S5 Fig). Note, the gynoecium is a very complex structure with many different tissues. Therefore, we cannot exclude that the changes in expression levels in specific tissues of the gynoecium are not well reflected in this assay. Therefore, we performed an *in situ* mRNA hybridization on *ARR1* in wild-type and *spt* gynoecia, because *ARR1* transcript abundance showed the most conspicuous reduction in dissected *spt* gynoecia. In wild-type, *ARR1* transcripts are present in the CMM, ovule primordia, and in the style region, overlapping with *SPT* expression pattern (Fig 3G and 3H; S6 Fig). However, in the *spt* mutant, *ARR1* messenger was either not detected or detected at very reduced levels, suggesting that SPT was required for *ARR1* expression (Fig 3I and 3J). These results support a role for SPT positively regulating the cytokinin signaling pathway by modulating the expression of at least two type-B *ARR* genes, *ARR1* and *ARR12*. On the other hand, since the qRT-PCR experiment did not show a reduction of *ARR10* in the *spt* mutant, we cannot conclude that it is also positively regulated by SPT as *ARR1* and *ARR12*. Therefore, it is highly likely that SPT affects, besides *ARR1* and *ARR12*, other components of the cytokinin signaling pathway.

The *ARR1* promoter fragment contains a G-box, a *cis*-regulatory motif targeted by bHLH transcription factors (as SPT) for gene regulation [39,56,57]. *ARR10* and *ARR12* have also bHLH binding motifs in their promoters, but do not have the G-box version. It has been reported that SPT binds only to the G-box version [39,57]. Therefore, the positive regulation by SPT on *ARR12* expression is most likely indirect or performed by a complex where SPT participates.

To determine whether SPT is able to positively regulate *ARR1* directly, we performed luciferase transient reporter assays in *Nicotiana benthamiana* leaves [58,59]. We observed that transiently expressed SPT protein was able to activate an *ARR1*::*LUC* reporter construct (Fig 3K). To further determine whether this regulation could be due to direct binding to *ARR1* regulatory regions in the DNA, we performed chromatin immunoprecipitation assays followed by qPCR (ChIP-qPCR) using *35S*::*SPT-HA* and wild-type *Arabidopsis* inflorescence tissue (Fig 3L). When compared to wild-type, ChIP-qPCR results from the *35S*::*SPT-HA* line showed a significant enrichment of the *ARR1* promoter fragment that contains the G-box, reported to be targeted by SPT [39,56,57].

In summary, all these results together are consistent with SPT being able to activate *ARR1* expression in gynoecia, possibly in a direct manner. Furthermore, SPT likely regulates *ARR12* as well, but in an indirect manner. Moreover, this regulation would also explain the lack of response to exogenous cytokinin application in *spt* and in the *arr1 arr12* double mutant and, at least partly, the reduction of cytokinin-induced signal response in the CMM and septa primordia in *spt* gynoecia. However, we cannot discard the possibility of indirect effects of SPT on type-B *ARR* expression and it is highly likely that SPT affects, besides *ARR1* and *ARR12*, also other components of the cytokinin signaling pathway.

### Cytokinin signaling activates an auxin biosynthetic gene in a SPT-dependent manner

The next question was whether and how the cytokinin signaling pathway interacts with the auxin pathway in gynoecia. It has been previously described that the cytokinin signaling pathway and *SPT* can interact with several genes in the auxin signaling pathway [13,28,31,38,39,55,60]. We therefore explored whether interactions between them were also taking place during the formation of medial tissues in the gynoecia.

**Fig 4:**
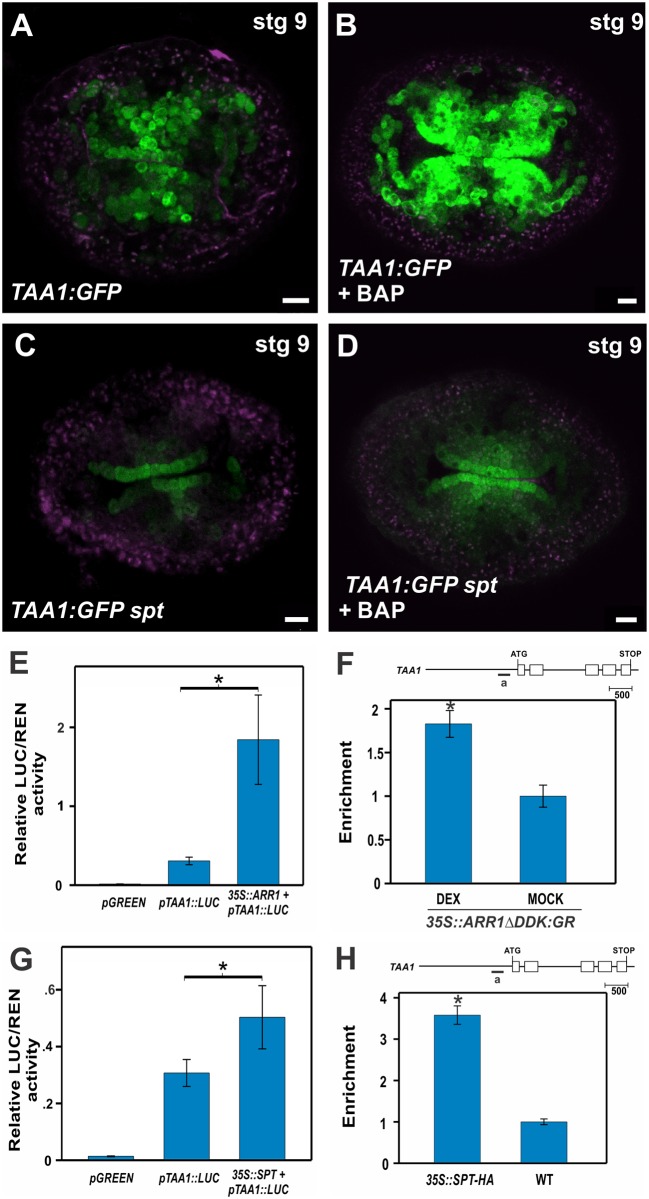
Cytokinin signaling activates the auxin biosynthetic gene *TAA1* in a SPT-dependent manner. **(A**, **B)** Expression of the translational fusion *TAA1*::*GFP-TAA1* in a transverse section of a stage 9 wild-type gynoecium that either received mock **(A)** or BAP treatment for 48 hours **(B)**. **(C, D)** Expression of the translational fusion *TAA1*::*GFP-TAA1* in a transverse section of a stage 9 *spt-12* gynoecium that received mock **(C)** or BAP treatment for 48 hours **(D)**. **(E)** Luciferase reporter assay in *N*. *benthamiana* leaves co-transformed with *35S*::*ARR1* and *pTAA1*::*LUC*. Ratio of LUC/REN activity. (**F**) ChIP experiments against the *TAA1* promoter region (indicated by “a” in the scheme above) using an inducible *35S*::*ARR1ΔDDK*:*GR* line treated with dexamethasone or mock. *ACT2/7* served as a negative control. **(G)** Luciferase reporter assay in *N*. *benthamiana* leaves co-transformed with *35S*::*SPT* and *pTAA1*::*LUC*. Ratio of LUC/REN activity. **(H)** ChIP experiments against the *TAA1* promoter region (indicated by “a” in the scheme above) using a *35S*::*SPT-HA* line and wild-type. *ACT2/7* served as a negative control. Error bars represent the SD for the LUC assays based on three biological replicates. ChIP results of one representative experiment are shown; error bars represent the SD of the technical replicates. *P < 0.05 (LUC: Student-t test; qPCR: ANOVA). Scale bars: 10 μm (A-D).

Interestingly, *TCS*::*GFP*, *SPT*, and the auxin biosynthesis gene *TRYPTOPHAN AMINOTRANSFERASE OF ARABIDOPSIS 1* (*TAA1*) are co-expressed in the CMM (Fig 4) [36,61], and mutant combinations of *TAA1* with its paralog *TAR2* (*TRYPTOPHAN AMINOTRANSFERASE RELATED 2*) resulted in plants with severely affected gynoecium development, indicating the importance of local auxin biosynthesis for correct gynoecium morphogenesis [61].

To test whether cytokinin had an effect on *TAA1* expression, we applied cytokinin to *TAA1*:*GFP* inflorescences and observed a strong increase in the GFP signal in the medial domain of stage 8 and 9 gynoecia, indicating that cytokinin induces *TAA1* expression (Fig 4B). Moreover, a microarray data meta-analysis has also identified *TAA1* as a cytokinin-responsive gene [62].

We then aimed at exploring the molecular mechanism involved in this induction. Since type-B ARRs are important positive regulators of cytokinin transcriptional response, we investigated whether ARR1 could activate *TAA1*. We found that transiently expressed ARR1 is able to activate a *TAA1*::*LUC* reporter construct in transient assays in *N*. *benthamiana* leaves (Fig 4E). We next performed ChIP-qPCR assays using the dexamethasone (DEX) inducible glucocorticoid receptor (GR) fusion line *35S*::*ARR1ΔDDK-GR*. In this line, upon DEX induction, ARR1 is constitutively active in the absence of cytokinin [63,64]. ChIP-qPCR results from DEX-treated *35S*::*ARR1ΔDDK-GR* inflorescences, when compared to those mock-treated, showed significant enrichment of a *TAA1* promoter fragment that contains various type-B ARR binding sites (Fig 4F), consistent with ARR1 directly regulating *TAA1*. This strongly suggests that one of the outputs of the cytokinin signaling pathway is to activate the auxin biosynthetic pathway and that ARR1 is a hub connecting the cytokinin signaling and auxin pathway. Our results further substantiate previous reports suggesting the connection between ARR1 and auxin biosynthesis [65].

Given the fact that SPT enables cytokinin responses in the early gynoecium, and that it likely activates *ARR1* directly, we evaluated *TAA1* response to cytokinin in *spt* mutants. As expected, when *SPT* is mutated, gynoecium cytokinin-dependent *TAA1*::*GFP* induction is abolished (Fig 4D). We also analyzed *TAA1*:*GFP* expression in untreated *spt* gynoecia, and observed a moderate reduction in GFP signal (Fig 4C), suggesting that SPT is able to activate *TAA1*, although additional regulators might contribute to *TAA1* expression in developing gynoecia. Interestingly, besides being required for the cytokinin induction of *TAA1* expression, based on our luciferase transient reporter assays (Fig 4G) and ChIP-qPCR experiments (Fig 4H), SPT seems to directly regulate *TAA1* expression by recognizing a *cis*-motif present within the *TAA1* promoter (Fig 4H).

In summary, these results indicate that both cytokinin and SPT can activate *TAA1* at the medial domain of the ovary, probably in a cooperative fashion, integrating a regulatory node for correct cytokinin signaling and auxin biosynthesis in the medial tissues of the gynoecia. This *TAA1* activation might be mediated by phosphorylated type-B ARRs and SPT, possibly as direct regulators, as the ARR1 and the SPT activation of *TAA1* and ChIP experiments suggest, though indirect regulation cannot be discarded.

### The auxin transporter *PIN3* is coordinately activated by cytokinin and SPT

**Fig 5:**
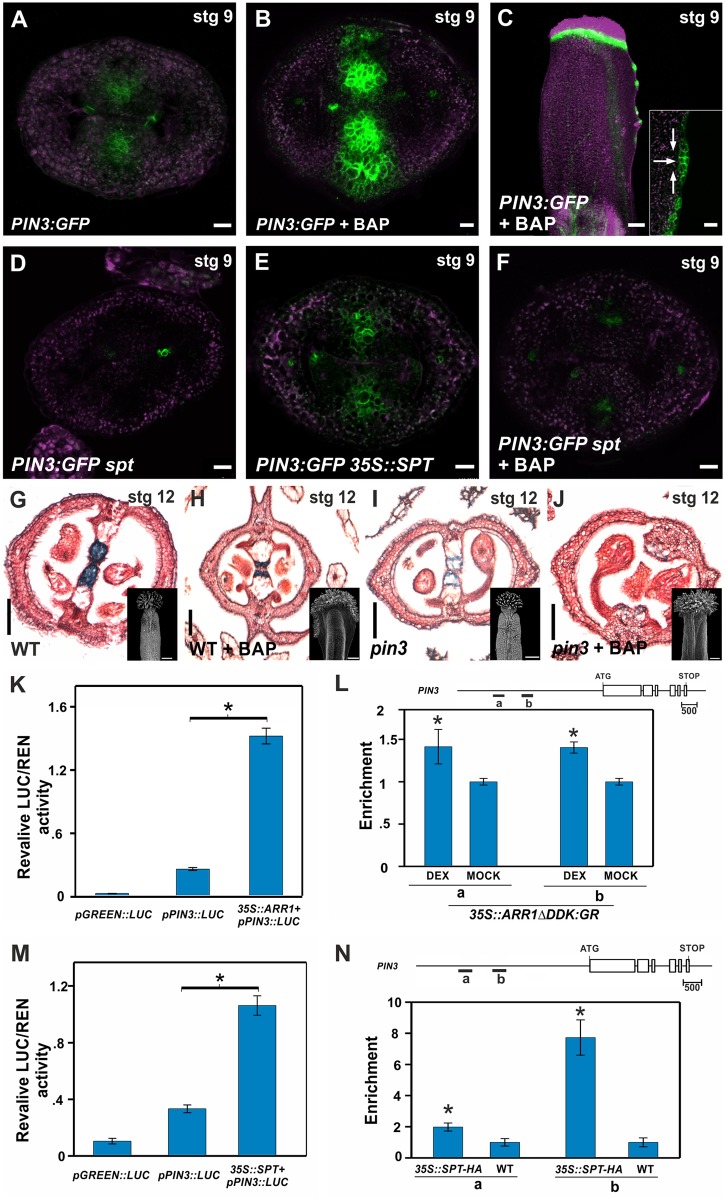
The auxin transporter *PIN3* is coordinately activated by cytokinin and SPT. **(A-C)** PIN3 expression in stage 9 *PIN3*::*PIN3-GFP* gynoecia that either received mock (**A,** transverse section) or BAP treatment for 48 hours (**B**, transverse section and **C**, longitudinal view). The inset in **(C)** shows a magnified view of the proliferating tissue. Arrows indicate the possible auxin flow. **(D-F)** PIN3 expression in transverse sections of stage 9 *PIN3*::*PIN3-GFP* gynoecia in *spt-2* **(D)**, *35S*::*SPT* **(E)**, and in *spt-2* treated for 48 hours with BAP **(F)**. **(G-J)** Transverse sections of stage 12 gynoecia of wild-type **(G, H)** and *pin3-4* **(I, J)**. Gynoecia phenotypes after three to four weeks of mock **(G, I)** or BAP treatment for five days **(H, J)**. Insets show a scanning electron microscopy image of the gynoecium. **(K)** Luciferase reporter assay in *N*. *benthamiana* leaves co-transformed with *35S*::*ARR1* and *pPIN3*::*LUC*. Ratio of LUC/REN activity. **(L)** ChIP experiments against the *PIN3* promoter regions (indicated by “a” and “b” in the scheme above) using an inducible *35S*::*ARR1ΔDDK*:*GR* line treated with dexamethasone or mock. *ACT2/7* served as a negative control. **(M)** Luciferase reporter assay in *N*. *benthamiana* leaves co-transformed with *35S*::*SPT* and *pPIN3*::*LUC*. Ratio of LUC/REN activity. **(N)** ChIP experiments against the *PIN3* promoter regions (indicated by “a” and “b” in the scheme above) using a *35S*::*SPT-HA* line and wild-type. *ACT2/7* served as a negative control. Error bars represent the SD for the LUC assays based on three biological replicates. ChIP results of one representative experiment is shown and the error bars represent the SD of the technical replicates. *P < 0.05 (LUC: Student-t test; qPCR: ANOVA). Scale bars: 10 μm (A-F), 100 μm (G-J, G-J insets). Ovule primordium (op).

Intriguingly, the expression of *TAA1* at the medial tissues of the ovary did not coincide with the expression of the auxin reporter *DR5rev*::*GFP*, which was not detected in these tissues (CMM, septa primordia, septum and transmitting tract) (S7A–S7D Fig). One possible explanation for this discrepancy is that the auxin synthesized by *TAA1* is transported outside these tissues by PIN auxin efflux transporters [66]. To determine whether this was the case, we analyzed the expression pattern of different GFP reporters for *PIN* genes (*PIN1*, *3*, *4* and *7*) in the medial region of the ovary of wild-type gynoecia and observed that *PIN1*:*GFP* and *PIN3*:*GFP* were expressed in the medial domain (S7 and S8 Figs). Since *pin1* mutants do not produce flowers [67], we focussed most of our analyses on *PIN3*. *PIN3*:*GFP* signal was detected in the CMM, septa primordia, septum and transmitting tract (Fig 5A; S7F and S8K–S8T Figs).

Given that cytokinin signaling was also detected there, we investigated whether cytokinin was influencing *PIN3* expression. Interestingly, when cytokinin was applied, the *PIN3*:*GFP* reporter was strongly induced in the medial domain of the ovary, while it seemed to be localized in a non-polar fashion in the cells (Fig 5B; S9M–S9O Fig). This clearly indicated that *PIN3* is responsive to cytokinin in these tissues. Accordingly, we observed a similar induction of *PIN3* and *PIN1* expression by cytokinin in the ectopic outgrowths produced from the medial region of the ovary (Fig 5C; S9N Fig). In these tissues, PIN3:GFP and PIN1:GFP were polarly localized towards the emerging outgrowths (Fig 5C; S9X Fig), which also showed a high *DR5rev*::*GFP* signal at their tips [7].

To determine whether *PIN3* is relevant for the overproliferation of the medial tissue after cytokinin treatment, we applied cytokinin to *pin3* inflorescences (*pin1* could not be tested due to the lack of inflorescences). If *PIN3* is required, the ectopic outgrowths would not be produced. Indeed, a minor ectopic medial outgrowth was observed in cytokinin-treated *pin3* gynoecia (S10D Fig) and only apical-basal defects were detected in 78.2% of the cases (i.e., alterations in the size of the ovary, gynophore and style with respect to each other) (n = 330) (Fig 5G–5J and insets) [32]. This suggests that observed medial tissue responses to exogenously applied cytokinin require a functional *PIN3*.

To explore the role of PIN3 in medial tissue development, we analyzed thin sections of *pin3* untreated gynoecia and observed mild alterations in transmitting tract development (Fig 5G–5J), characterized by reduced blue staining of the cells. A possible explanation for the *pin3* mild phenotype in the medial tissue is that the related PIN7 can partially compensate for the PIN3 function, as it has been reported in other developmental programs [68]. Accordingly, the double *pin3 pin7* mutant has severe floral defects and none of the gynoecia form correctly (S10E–S10H Fig) [66]. However, we were not able to detect PIN7 signal in wild type ovaries, perhaps due to low signal of the reporter line. On the other hand, another explanation is that PIN1 partially compensates for the PIN3 loss, because PIN1 signal is clearly detected in the medial tissues and the reporter line responds to cytokinin (S8 and S9 Figs).

To obtain insights about the possible molecular mechanism by which cytokinin activates *PIN3* expression at the medial domain, and since type-B ARRs are important effectors of cytokinin signaling, we explored whether type-B ARR activity could be involved, using ARR1 to test this. We found that ARR1 was able to activate *PIN3* in luciferase transient reporter assays (Fig 5K). Moreover, ChIP-qPCR data were compatible with the possibility of ARR1 directly activating *PIN3* expression in the medial domain of the gynoecia (Fig 5L). The data suggests that ARR1 is able to bind two regions within the PIN3 promoter containing putative *cis*-regulatory motifs for type-B ARR transcription factors.

Next, we investigated whether SPT could also regulate *PIN3* expression and whether the *PIN3* cytokinin response was SPT-dependent. Expression of *PIN3* in the CMM and septa primordia appears to be dependent on *SPT* given that no *PIN3* expression in these medial tissues was observed in a *spt* mutant background (Fig 5D; S9E–S9H Fig), whereas it increased when *SPT* was constitutively expressed (Fig 5E; S9I–S9L Fig). qRT-PCRs using dissected gynoecia also showed a decrease in *PIN3* expression in a *spt* background and an increase in a *35S*::*SPT* background (S9W Fig). Moreover, induction of *PIN3* expression by cytokinin is also dependent on the presence of *SPT*, as no PIN3 cytokinin-dependent activation was seen in *spt* ovaries (Fig 5F; S9P–S9R Fig). Though indirect regulation cannot be discarded, luciferase transient reporter assays (Fig 5M) together with ChIP-qPCR data (Fig 5N) were consistent with direct regulation of *PIN3* by SPT. Specifically, and based on our results, SPT bound to two regions (`a`and preferentially `b`) within the *PIN3* promoter (Fig 5N).

In summary, all these results together support that SPT and the cytokinin-signaling pathway, probably in a cooperative fashion, are connected to auxin biosynthesis and auxin transport at the medial domain of the ovary.

Cytokinin signaling repressors are expressed in the lateral domain After finding that the localization of cytokinin signaling in the medial domain depends on *SPT*, we still wondered whether other factors could be repressing cytokinin signaling in the lateral domain. Besides barely detecting *TCS*::*GFP* expression at the lateral domain, we also had observed that exogenous cytokinin treatments could not activate this marker in the lateral domain (presumptive valves). Moreover, TCS::GFP signal could also not be detected in the lateral domain of *35S*::*SPT* gynoecia, not even when cytokinin was added to this line (S11 Fig). Finally, the outgrowths promoted by cytokinin treatments were observed to arise from the medial and not the lateral domain of the gynoecium. Together, these observations indicate that gynoecia lateral tissues (presumptive valves) are not responsive to the exogenously applied cytokinin or ectopic *SPT* expression, and suggest that repression is taking place in these tissues.

**Fig 6:**
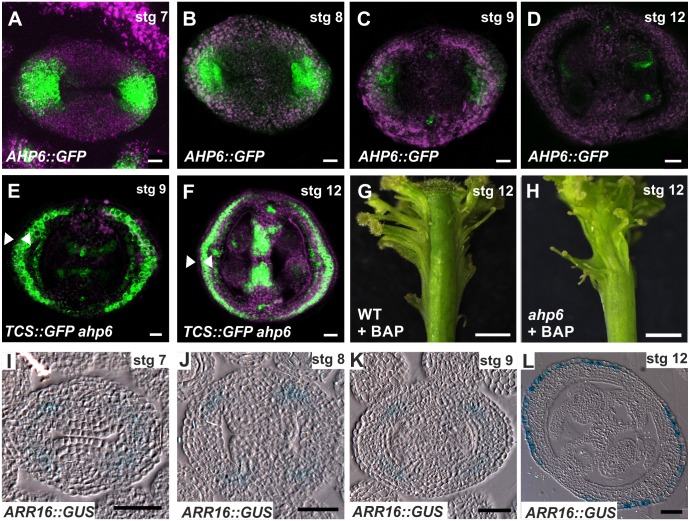
The cytokinin signaling repressors *AHP6* and *ARR16* likely block cytokinin responses in lateral tissues. **(A-D)** Expression of the transcriptional reporter *AHP6*::*GFP* in transverse sections of stage 7, 8, 9, and 12 gynoecia. **(E, F)** Expression of the cytokinin response reporter *TCS*::*GFP* in transverse sections of stage 9 and 12 gynoecia in an *ahp6-1* mutant background. Arrowheads indicate the absence of GFP signal in the epidermis of the valves. **(G, H)** Phenotypes of wild-type (G) and *ahp6-1* (H) gynoecia one week after receiving BAP treatment for two weeks. **(I-L)** Expression of the transcriptional reporter *ARR16*::*GUS* (type-A *ARR*) in transverse sections of stage 7, 8, 9, and 12 gynoecia. Scale bars: 10 μm (A-C, E), 20 μm (D, F), 1 mm (G, H), 100 μm (I-L).

As mentioned in the Introduction, some components of the cytokinin signaling pathway execute a repressing effect on cytokinin-dependent outputs. We therefore wondered whether these repressors were present in the lateral domain of the gynoecium. The gene *AHP6* encodes a histidine phosphotransfer protein that inhibits cytokinin signaling responses [69] and it has been also shown to participate in auxin-cytokinin communication (i.e., it is induced by auxin) [41,69,70]. In gynoecia, *AHP6*::*GFP* reporter activity is strong in the lateral domains of stage 7, 8, and 9 gynoecia (Fig 6A–6D), suggesting that *AHP6* is negatively regulating cytokinin signaling in the valves. As mentioned above, the *TCS*::*GFP* reporter is not active in the lateral domains of gynoecia (Fig 1). However, this marker was ectopically active in the lateral domains of *ahp6* gynoecia (Fig 6E and 6F). Moreover, repeated cytokinin applications to *ahp6* inflorescences caused ectopic tissue proliferation in a radial pattern in the apical part of the gynoecium, a morphological effect not seen in wild-type treated gynoecia (Fig 6G and 6H).

Interestingly, we also observed that the *GUS*-reporter construct (*ARR16*::*GUS*) for the type-A *ARR16* gene, which encodes a regulatory protein that negatively regulates cytokinin signaling pathway responses [13], was active in the lateral domain of stage 7, 8, 9, and 12 gynoecia (Fig 6I–6L).

**Fig 7:**
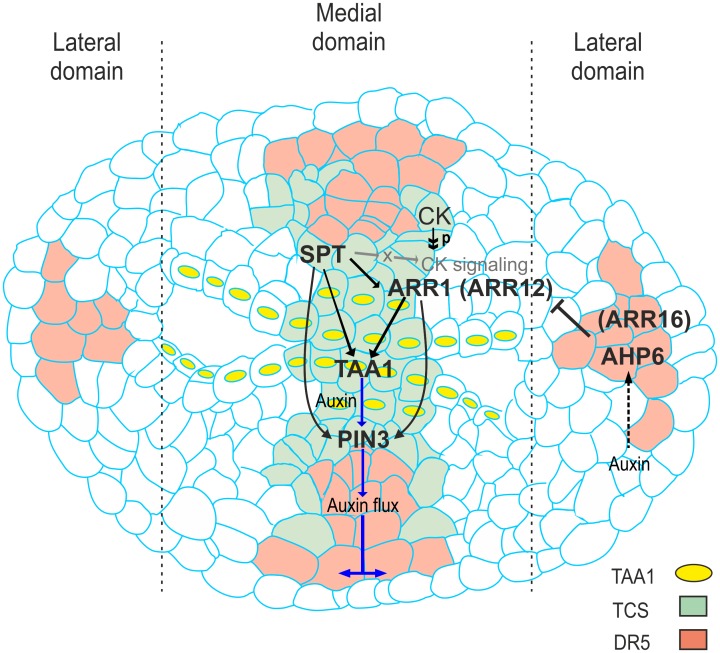
Model of the regulatory network in early gynoecium development integrating SPT, cytokinin signaling, auxin biosynthesis, and auxin transport. Model of the regulatory network in early gynoecium development. This regulatory network integrates the results that SPT, an important player of gynoecium development, enables cytokinin signaling in the medial domain of the young gynoecium by activating the transcription of type-B *ARR* genes (at least *ARR1* and *ARR12;* likely *ARR1* directly and *ARR12* indirectly), which proteins become active upon phosphorylation because of a phosphorelay cascade initiated when cytokinin is present, and then together activate auxin biosynthesis (*TAA1*) and transport important (*PIN*) for growth. It is likely that SPT also affects other components of the cytokinin signaling pathway (indicated by gray arrows). Solid black arrows indicate a positive regulation and a T-bar indicates a repression function, a broken black arrow indicates possible positive regulation by auxin, a double arrowhead indicates phosphorylation, purple arrows indicate possible auxin flow; CK, cytokinin; P, phosphate group.

Altogether, these data support a scenario in which the cytokinin signaling pathway is negatively regulated in the lateral domain of the gynoecium by *AHP6*, and perhaps by *ARR16* as well. This negative regulation can confine cytokinin signaling to the medial domain of the gynoecium (Fig 7).

**Fig 8:**
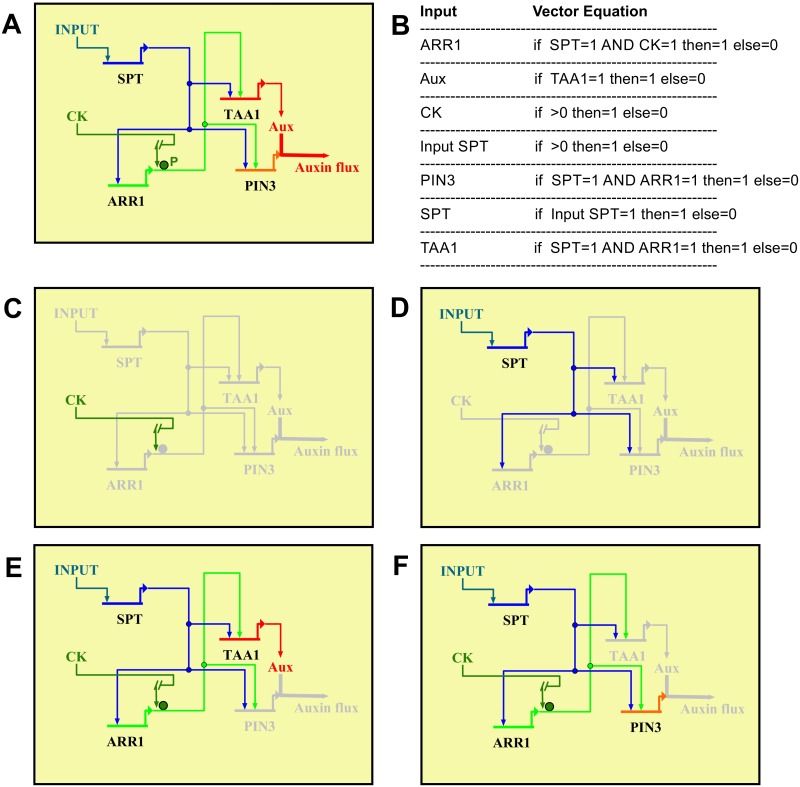
Dynamic GRN Boolean model active during early gynoecium development. **(A)** The topology of the Gene Regulatory Network (GRN) model visualized using the computational and graphical platform BioTapestry [71]. Regulatory relations among genes are based on the experimental evidence (this work). The two coherent feed-forward subcircuits are formed by starting with *SPT* regulating *ARR1* and both together regulate *TAA1*, and also both together regulate *PIN3*. *TAA1* also has other positive regulators, but for simplicity only *SPT* and *ARR1* are depicted. *ARR1* is depicted, but *ARR12* is likely part of the network too. CK: cytokinin; Aux: auxin; P: phosphorylation. **(B)** All regulatory interactions were fed to the computational tool GeNeTool [72] to create the Boolean vector equations and for modeling of the GRN. The coherent feed-forward subcircuits are both configured as an AND-gate, i.e., cooperate regulation. Boolean output for gene active = 1 and for gene inactive = 0. **(C-F)** Alterations of the topology of the GRN model after perturbations. Boolean output was calculated by GeNeTool for each perturbation and visualized with BioTapestry. Gray color of lines and genes means it is inactive. The GRN is affected after the following perturbation experiments: *SPT* off **(C)**, CK signaling off (D), *PIN3* off **(E)**, and *TAA1* off **(F)**. In all cases the GRN is altered, which predicts that gynoecium development will also be altered, and this happens in the mutants, validating our GRN model.

A dynamic GRN Boolean model during early gynoecium development In this work, we found that SPT enables cytokinin signaling at the medial domain. Moreover, both SPT and cytokinin signaling, probably together, positively regulate auxin biosynthesis and transport genes in this domain. Based on these findings we developed a preliminary gene regulatory network (GRN; Figs 7 and 8) that acts during early gynoecium development. To verify that this network fits the experimental data, we made a GRN Boolean model using the computational tools BioTapestry [71] and GeNeTool [72] (Fig 8A and 8B), and we confirmed that the wiring of this network gives a stable output (i.e., fixed steady state for each gene) (Fig 8A). Note, we modeled *TAA1* and *PIN3* regulation by SPT and ARR1 in a cooperative manner (i.e., meaning that both are necessary). The possible cooperative regulation could be through the formation of a protein complex. Support for this is the observation that SPT and ARR1 bind to the same fragments of the *TAA1* and *PIN3* promoters in ChIP assays. We already explored whether these two transcription factors interact directly. However, we could not detect any protein-protein interaction in yeast two-hybrid (between SPT and ARR proteins) nor in a bimolecular fluorescence complementation assays (between SPT and ARR1) (S12 Fig). Nevertheless, SPT and ARR1 could be part of a higher-order complex where both factors are present but do not interact directly. Note, cytokinin should be as well present, to start the phosphorelay cascade that finally leads to phosphorylation of the type-B ARR proteins so that they are functional. Moreover, *in silico* perturbations of the regulatory links in our model produces the observed phenotypes (Fig 8C–8F). The experimental evidence supports direct regulatory links between the genes, but the value of the identified GRN holds even though if regulatory links would be indirect. The topology of the network presents interesting features. For example, the regulatory interactions between *SPT*, *ARR1*, and *TAA1* as well as between *SPT*, *ARR1*, and *PIN3* are wired as coherent feed-forward subcircuits [73,74]. This type of subcircuit has been detected in other plant developmental processes (e.g., [75–77]), and can integrate upstream spatial regulatory inputs, cause high expression of the target gene, and temporal delay in switching the target gene on or off [73,74].

## Discussion

In angiosperms, the correct patterning and morphogenesis of the gynoecium is an essential developmental program for the formation of reproductive tissues and, thus, the reproductive success of the plant. Research from many groups led to the identification of key regulatory genes governing gynoecium development (reviewed in: [1,3,78–80]). Several lines of evidence have also highlighted the importance of hormones during gynoecium development (reviewed in: [1,5,6,25,81,82]). However, the interaction between the gene regulatory layers and hormonal pathways, the mechanisms that determine different hormone responsive and non-responsive regions, and the interaction between hormonal pathways in the medial tissues of the ovary of the young gynoecium, are topics not explored in depth.

In our study we addressed these questions from the cytokinin signaling point of view. Cytokinin signaling is localized in the medial domain of the young gynoecium, and the triple type-B *arr* mutant phenotypes indicate that proper signaling is required for the correct formation of medial domain structures in the ovary, necessary for normal gynoecium and fruit development. Interestingly, *STM*, which can activate the cytokinin biosynthesis *IPT* genes, is expressed at the CMM [15,83], and inducible repression of *STM* causes carpel fusion defects, reduced CMM development, a reduction in placenta and ovule number, and even a complete absence of the gynoecium [84]. Conversely, increased cytokinin levels in the gynoecium causes a larger placenta and more ovules [11], and increased replum width [7], further supporting an important role for cytokinin in early gynoecium development.

The bHLH transcription factor SPT is key for early gynoecium development [45–47]. Previous reports showed that members of the bHLH transcription factor family are related to hormone signaling pathways (e.g., [85–89]). In our study, most importantly, we have identified that SPT enables cytokinin response at the medial domain, thereby stimulating meristematic activity in this domain. The lack of cytokinin signaling observed in *spt* explains why the CMM and septa primordia of analyzed stage 8 *spt* gynoecia contain fewer cells, and why, at later stages, less ovules are formed in the mutant than in the wild-type [46]. SPT may act through type-B ARRs, and results support a direct regulation of *ARR1* by SPT. Furthermore, results also suggest regulation of *ARR12* by SPT, though, this regulation is likely indirect because, though its promoter contains bHLHs motifs, a true G-box bound by SPT was not detected [39,57]. On the other hand, since the qRT-PCR experiment did not show a reduction of *ARR10* in the *spt* mutant, we cannot conclude that it is also positively regulated by SPT as *ARR1* and *ARR12*. If SPT does not regulate *ARR10*, it would be most likely regulating additional components of the cytokinin signaling pathway, because, though the *arr1 arr12* double mutant has a reduced response to exogenous cytokinins, it does not present a severe mutant gynoecium phenotype. Still, the promoter regions of *ARR10* contain bHLH binding motifs, suggesting that it could be regulated by another bHLH transcription factor.

Moreover, we identified close links between the cytokinin and auxin pathways at the medial domain of the gynoecium. Both SPT and ARR1 regulate *TAA1* and *PIN3*, components of the auxin pathway, in the medial domain, probably causing a PIN3-dependent auxin flux away (auxin drainage) from the gynoecium centre towards the repla and the valves. The regulation of *TAA1* and *PIN3* by SPT and ARR1 may be cooperative. Most likely, auxin is directed afterwards to the apical and/or basal part of the gynoecium, where it can flow from the base to the top and back, as recently proposed in the ‘reverse fountain’ model [35]. Auxin drainage would be important for growth and, furthermore, auxin flow in the lateral domains (presumptive valves) would prevent them from obtaining medial domain identity [35]. Evidence for the importance of auxin transport comes from the observation of strong defects in medial domain development in the double mutant for the genes *REVOLUTA* (*REV*) and *AINTEGUMENTA* (*ANT*), where auxin transport was altered [36].

The cytokinin signaling repressors *AHP6* and *ARR16* are found at the presumptive valve tissues (lateral domain) and thereby, at least *AHP6*, restrict the high cytokinin signaling output that stimulates meristematic activity to the medial domain. This restriction of cytokinin signaling explains why no expansion of TCS signal from medial to lateral domain is observed in exogenous cytokinin treated gynoecia, in *35S*::*SPT* gynoecia, nor in cytokinin treated *35S*::*SPT* gynoecia.

The *ahp6* mutant gynoecia showed TCS::GFP signal in the valves and appeared to be more sensitive to cytokinin applications compared to wild-type, although the non-treated gynoecia of the mutant appeared normal, suggesting redundancy of this cytokinin restriction function. One thing we noticed is that the TCS::GFP signal in *ahp6* gynoecia did not extend to the epidermis of the valves. It has been reported that the epidermis is important for signaling [90]. Perhaps only in double or higher-order mutants for valve expressed cytokinin signaling repressors, gynoecial developmental defects can be observed. Another point of interest is that AHP6 is involved in the hormonal communication between auxin and cytokinin [41]. In vascular pattern formation, the auxin-induced cytokinin signaling repressor AHP6 is involved in the establishment of two mutually inhibitory domains [41]. In the SAM, AHP6 is also involved in establishing inhibitory fields, important for phyllotaxis [70]. It will be interesting in future studies to investigate if AHP6 is involved in establishing inhibitory fields also in the gynoecium. In principle, we already observed separate fields of cytokinin and auxin responses in the young gynoecium. It will also be interesting to investigate if it is the produced auxin induced by cytokinin in the medial domain of the gynoecium that then gets transported by cytokinin-induced PIN3 to the lateral domains to activate AHP6, or whether *AHP6* is under the control of the regulatory genes required for lateral tissue formation. Future experiments might provide further insights into how cytokinin controls the gynoecium and its impact on patterning.

Interestingly, a function for the *HEC* genes and *SPT* in SAM function was recently reported: *HECs* were shown to stimulate stem cell proliferation in a tissue-specific and *SPT*-dependent manner, suggesting that the relative levels of these transcription factors dictate the proliferative potential of stem cells [91]. A reduced SAM size was observed in *spt* mutant plants [91], which suggests that SPT function is also likely to be necessary for a positive cytokinin signaling output in the SAM. It would be interesting to explore other elements participating in the regulatory network in early gynoecium development, including the *HEC* genes, whose triple mutant has similar developmental defects in medial tissues to those observed in the *spt* mutant [92]. A recent study has already started to explore HEC–SPT function in style and stigma formation, demonstrating positive regulation of auxin biosynthesis and transport, and likely repressing cytokinin signaling in the apical region of the gynoecium [55]. This indicates that the role of SPT in the style and stigma is different from that in the ovary.

Some of the effects of cytokinin application resemble phenotypes observed in polarity mutants (this work) [7,32,93,94]. It would be very interesting to further explore how the network here described interacts or even overlaps with known polarity cues or regulators such as *CRABS CLAW* (*CRC*) [46,95], *ETTIN* (*ETT*) [96], *REVOLUTA* (*REV*) [97], or *KANADI* (*KAN*) [98–101].

The gynoecium is a key component of the success of the angiosperms [102,103], which comprise over 300,000 species on earth. Here we showed, for a Brassicaceae family member, that cytokinin signaling is necessary for its correct development and, therefore, for reproductive competence. Interestingly, the presence of the bHLH transcription factor *SPT*, cytokinin signaling and auxin biosynthesis genes, and *PIN* orthologs in basal angiosperms [57,104–108], suggests that these genes already could have a function in gynoecium development in early flowering plants. Future work should shed light on how and when this network emerged.

## Materials and methods

### Plant materials and growth conditions

Seeds were obtained for *spt-2* (CS275*)*, *arr1-3* (CS6971), *arr10-5* (CS39989), *arr12-1* (CS6978), *arr1-3 arr10-5* (CS39990), *arr1-3 arr12-1* (CS6981), *arr10-5 arr12-1* (CS39991), *arr1-3 arr10-5 arr12-1* (CS39992), and *DR5rev*::*GFP* (CS9361) from the Arabidopsis Biological Resource Center (Ohio State University, Columbus), *TCS*::*GFP* from Bruno Muller, *pSPT-6253*:*GUS* from David Smyth, *spt-12*, *35S*::*SPT*, and *35S*::*SPT-HA* from Karen Halliday, *pin3-4* and *pin3 pin7* from Eva Benková, *PIN3*::*PIN3-GFP* and *TAA1*::*GFP-TAA1 spt-12* from Lars Østergaard, *PIN1*::*PIN1-GFP* and *PIN7*::*PIN7-GFP* from Luis Herrera-Estrella, *PIN4*::*PIN4-GFP* from Elena Alvarez-Buylla, *TAA1*::*GFP-TAA1* from Anna Stepanova, *AHP6*::*GFP* from Ykä Helariutta, *TCS*::*GFP* in the *ahp6-1* background from Teva Vernoux, *35S*::*ARR1ΔDDK-GR* from Takashi Aoyama, and *ARR16*::*GUS* from Takeshi Mizuno. *Arabidopsis thaliana*, *Nicothiana tabacum*, and *Nicotiana benthamiana* were grown in soil under normal greenhouse conditions or in a growth chamber (~22°C, long day light regime).

### Cytokinin treatments

Inflorescences were treated with cytokinin 6-Benzylaminopurine (BAP) as previously described [32]. In summary, one week after bolting, BAP solution drops were placed on the inflorescences once a day for 2 (48-hour period) or 5 to 10 (repeated applications) consecutive days. To observe ectopic outgrowths from the medial domain of the pistil, a BAP treatment for five days is given and after three to four weeks observations are made. The BAP solution contains 100 μM 6-benzylaminopurine (BAP; Duchefa Biochemie) and 0.01% Silwet L-77 (Lehle Seeds) in distilled water. Mock treatments contained only 0.01% Silwet L-77 in distilled water. All treated plants with their respective controls were cultivated simultaneously under the same growth conditions.

### Gene expression analysis

For qRT-PCR analysis, stage 8–10 gynoecia or inflorescence with only floral buds were collected and total RNA was extracted using TRIzol (Invitrogen). After DNAse I (Invitrogen) treatment, cDNA was prepared using SuperScript III Reverse Transcriptase (Invitrogen) according to manufacturer’s instructions and using reverse specific primers for each of the corresponding genes under test (S1 Table). The cDNA was analyzed in an ABI PRISM 7500 sequence detection system (Applied Biosystems) with SYBR Green Master Mix (Applied Biosystems) according to the manufacturer’s instructions. Three biological replicates and four technical replicates were done for each assay. Data was analyzed using the 2^-ΔΔCT^ method [109]. Target gene expression levels were normalized to *ACTIN2/7*. Primer sequences are listed in S1 Table.

### *In situ* hybridization

*In situ* hybridization was carried out as previously described [110]. The template for the DIG-labeled antisense and sense probe synthesis for *ARR1* mRNA was generated by PCR using specific primers (S1 Table) and inflorescence wild-type cDNA. The resulting PCR fragment was purified, sequenced and used as template to transcribe the antisense probe with the T7 RNA polymerase (Promega) and the sense probe with the SP6 polymerase (Promega).

### ChIP assay

ChIP experiments were performed as previously described [111]. Between 0.5 g and 1 g of inflorescences were collected for each experiment. The *35S*::*SPT-HA* homozygous transgenic line [112] and wild-type L*er* (as relative control) were used for ChIP on SPT. A monoclonal mouse anti-HA (Sigma; H3663) (2 μg per sample) was used to immunoprecipitate SPT-HA complexes. We additionally tested *35S*::*SPT-HA* line ChIP enrichment with no anti-HA antibody as a preliminary test, to ensure specificity of the ChIP reaction. ChIP assays for ARR1 were performed using the DEX (dexamethasone)-inducible *35S*::*ARR1ΔDDK-GR* line [64] after DEX induction. Mock treated plants were employed as controls. In short, DEX-treated *35S*::*ARR1ΔDDK-GR* inflorescences were collected 24 hours after two DEX applications (each one separated by 12 hours) with 10 μm DEX solution with 0.015% Silwet in distilled water, frozen in liquid nitrogen, and stored at -80°C till enough material was collected. Glucocorticoid Receptor alpha polyclonal antibody (Thermo Scientific; PA1-516) (2 μg per sample) was used to immunoprecipitate ARR1ΔDDK-GR complexes. Results from qPCR experiments were analyzed using the 2^-ΔΔCT^ method [109]. Each biological sample was assayed for relative enrichment with respect to its input sample (fragments were normalized using ACTIN2/7). Binding was concluded if PCR enrichment was detected in at least three out of five independent biological replicates. Primers used for ChIP-qPCR analysis are listed in S1 Table.

### Luciferase activity assay

Promoter regions of *PIN3* (4.3 kb, -4310 to ATG), *ARR1* (2.1 kb, -2116 to ATG), and *TAA1* (2 kb, -2047 to ATG) were amplified from *Arabidopsis* Col-0 genomic DNA with specific primer pairs (S1 Table), cloned into pGEM-T vector (Promega), digested with SmaI and NcoI restriction enzymes, and ligated into pGREEN-LUC [59] to generate *pPIN3*::*LUC*, *pARR1*::*LUC*, and *TAA1*::*LUC* reporters, respectively, for transient expression assays in *N*. *benthamiana* leaves. The *35S*::*SPT* effector construct that was used in the coinfiltrations with the corresponding *LUC* reporters, was generated by transferring the *SPT* ORF into the pEARLY100 vector [113] through Gateway reactions, which was previously cloned in the pDONR221 vector (Invitrogen). The *35S*::*HA-ARR1* has been previously described [114]. The transient Luciferase expression assays were performed by transient transformation of *N*. *benthamiana* leaves by Agrobacterium infiltration, which was performed as previously described [115] with minor modifications [58]. At least three plants at the same developmental stage were used for each treatment, and the experiments were repeated at least three times.

Tissue preparation and confocal microscopy analysis To observe fluorescence signal, gynoecia were dissected and observed as previously described [116]. In summary, gynoecia were observed longitudinally or cut transversely using a scalpel and mounted in glycerol. Propidium iodide (PI; Fluka), 50 μM PI for 30–60 seconds, was used as counterstain. Imaging was done using a LSM 510 META inverted confocal microscope (Carl Zeiss) with either a 20X or 40X air objective. GFP was excited with a 488 nm line of an Argon laser and PI with a 514 laser line. GFP emission was filtered with a BP 500–520 nm filter and PI emission was filtered with a LP 575 nm filter.

Scanning electron microscope analysis Fresh tissue samples were visualized in a Zeiss scanning electron microscope EVO40 (Carl Zeiss) using the VPSE G3 or the BSD detector with a 15–20 kV beam.

### GUS analysis

Gynoecia were dissected and pre-fixed with cold acetone for 20 min, rinsed, and transferred into GUS substrate solution: 50 mM sodium phosphate pH 7, 5 mM K3/K4 FeCN, 0.1% (w/v) Triton X-100, and 2 mM X-Gluc (Gold BioTechnology, Inc). After application of vacuum for 5 min, *SPT*::*GUS* and *ARR16*::*GUS* samples were incubated at 37°C for 12 hrs.

### Histology

Tissues were fixed in FAE (3.7% formaldehyde, 5% glacial acetic acid and 50% ethanol) with vacuum (15 min, 4°C) and incubated for 60 min at room temperature. The material was rinsed with 70% ethanol and incubated overnight at 4°C, followed by dehydration in a series of alcohol solutions (70, 85, 95, and 100% ethanol) for 60 min each and embedded in Technovit as previously described [117]. Pictures were taken using a Leica DM6000B microscope coupled with a DFC420 C camera (Leica).

Transmitting tract analysis Transmitting tract staining was performed as previously described [118].

### Yeast two-hybrid assay

The *SPT* cDNA was cloned in the pENTR/D TOPO vector (Invitrogen), verified by sequencing, and introduced into the LexA DNA-binding domain vector (pBTM116c-D9) by Gateway LR recombination. The ARRs fused to the Gal4 activation domain in pACT2 (Clontech, Mountain View, CA, USA) are previously described [119]. Yeast transformations were performed as previously described [120] using the L40ccaU strain (MATa his3D200 trp1-901 leu2-3 112 LYS::(lexAop)4-HIS3 URA3::(lexAop)8-lacZ, ADE2::(lexAop)8- URA3 GAL4 gal80 can1 cyh2) [121]. The assay was done on SD-Gluc medium lacking Leucine, Tryptophan, and Histidine complemented with 3 mM 3-Amino-1,2,4-triazole. Interactions were scored after growing yeast at 25°C for 5 days.

### Bimolecular fluorescence complementation assay

*SPT* and *ARR1* coding sequences in Gateway entry vectors were recombined with pYFC43 and pYFN43 to generate C- and N-terminal YFP fusion constructs, respectively [122]. BiFC in young *N*. *tabacum* leaves was previously described [117]. YFP signal was assayed 3 days after infiltration using a confocal microscope.

### GRN Boolean model

The topology of the Gene Regulatory Network (GRN) model was visualized using the computational and graphical platform BioTapestry [71]. Regulatory relations among genes are based on the experimental evidence (this work). The two coherent feed-forward subcircuits are formed by starting with *SPT* regulating *ARR1* and both regulate *TAA1*, and also both regulate *PIN3*. *TAA1* also has other positive regulators, but for simplicity only *SPT* and *ARR1* are depicted. *ARR1* is depicted, but other type-B *ARR* genes are part of the network. All regulatory interactions were fed to the computational tool GeNeTool [72] to create the Boolean vector equations and for modeling of the GRN. The two coherent feed-forward subcircuits are both configured as an AND-gate. Boolean output for gene active = 1 and for gene inactive = 0. Alterations of the topology of the GRN model after perturbations were calculated by GeNeTool and visualized with BioTapestry.

## Supporting information

S1 Table Primer sequences used in this study. (PDF)

S1 Fig *SPT* expression during gynoecium development. (**A-F)** Expression of *SPT*::*GUS* during gynoecium development at stage 7, 8, 9, 10, 11, and 12, respectively. Scale bars: 20 μm (A-C), 40 μm (D-F).
(TIF)

S2 Fig Phenotypes of wild-type, single, double, and triple type-B *arr* mutant plants. Photos of plants of 73 days old of wild-type (Col-0), *arr1*, *arr10*, *arr12*, *arr1 arr10*, *arr10 arr12*, *arr1 arr12*, and *arr1 arr10 arr12*. Scale bar: 3 cm.
(TIF)

S3 Fig Transverse sections of stage 6–12 gynoecia of wild-type, single, double, and triple type-B *arr* mutants. Transverse sections of the ovary region of stage 6–12 gynoecia of wild-type, *arr1*, *arr10*, *arr12*, *arr1 arr10*, *arr10 arr12*, *arr1 arr12*, and *arr1 arr10 arr12*. The photo of the stage 12 gynoecium of the triple type-B *arr* mutant is an example of a section with an apparently normal transmitting tract. Scale bars: 100 μm.
(TIF)

S4 Fig SPT enables cytokinin response during gynoecium development. (**A, B)** Phenotypes of wild-type L*er* (A) and *spt-2* (B) gynoecia treated with BAP for 5 days. The photos were taken 3–4 weeks after the BAP treatment. In **(B)** an example is shown of a *spt-2* gynoecium presenting a minor effect to BAP in the replum outgrowth phenotype (only in 12.5% of the cases). (**C)** Phenotypes of wild-type Col-0 (left) and of *spt-12* (right) gynoecia treated with mock or BAP for 48 hours. The photos were taken 1 day after the BAP treatment. (**D, E, H, I)** Transverse sections of stigma/style region of gynoecia of wild-type L*er* (mock) **(D)** and *spt-2* (mock) **(H)**, and of 48 hours BAP-treated gynoecia of wild-type L*er* **(E)** and of *spt-2* **(I)**. (**F, G, J, K)** Transverse sections of the ovary region of gynoecia of wild-type L*er* (mock) **(F)** and *spt-2* (mock) **(J)**, and of 48 hours BAP-treated gynoecia of wild-type L*er* **(G)** and of *spt-2* **(K)**. Scale bars: 10 mm (A-C), 150 μm (D-K).
(TIF)

S5 Fig qRT-PCR of *ARR1*, *ARR10*, and *ARR12* in wild-type gynoecia. Expression analysis by qRT-PCR of *ARR1*, *ARR10*, and *ARR12* in wild-type dissected gynoecia. Error bars represent the SD based on three biological replicates.
(TIF)

S6 Fig *In situ* hybridization with sense-probe for *ARR1* in the gynoecium. **(A)** Negative control (sense probe) for the *in situ* hybridization of the type-B *ARR1* in a longitudinal section of a stage 12 gynoecium. Scale bar: 100 μm.
(TIF)

S7 Fig Expression of *DR5*::*GFP* and auxin efflux PIN transporters in the gynoecium. **(A-D)** Expression of the transcriptional auxin response reporter *DR5*::*GFP* line in transverse sections of wild-type gynoecia at stages 8, 9, 10, and 12. **(E-L)** Expression of PIN translational fusions with GFP in gynoecia at stage 9 and 12: *PIN1*::*PIN1-GFP* **(E, I)**, *PIN3*::*PIN3-GFP* **(F, J)**, *PIN4*::*PIN4-GFP* **(G, K)**, and *PIN7*::*PIN7-GFP* **(H, L)**. Scale bars: 10 μm (A-C), 20 μm (D-H), 50 μm (I-L).
(TIF)

S8 Fig PIN1 and PIN3 localization during gynoecium development. (**A-J)** The localization of *PIN1*::*PIN1-GFP* during gynoecium development at stage 7, 8, 9, 10, and 12 (Longitudinal view: **A-E**; top view at the apex: **F**; transverse section in the ovary: **G-J**). (**K-T)** The localization of *PIN3*::*PIN3-GFP* during gynoecium development at stage 7, 8, 9, 10, and 12 (Longitudinal view: **K-O**; top view at the apex: **P**; transverse section in the ovary: **K-T**). Scale bars: 10 μm (A-C, F-I, K-M, P-S), 20 μm (D, E, J, N, O, T).
(TIF)

S9 Fig PIN3 localization during gynoecium development in different backgrounds and upon cytokinin treatment. **(A-L)** Localization of *PIN3*::*PIN3-GFP* in transverse sections of gynoecia at stage 7, 8, 9, and 12 of wild-type **(A-D)**, *spt-2* **(E-H)**, and *35S*::*SPT* **(I-L)**. **(M-R)** PIN3 expression after 48 hours BAP treatment of stage 8, 9, and 12 gynoecia in wild-type **(M-O)** and *spt-2* **(P-R)**. **(S-V)** Longitudinal view of PIN3 expression in a wild-type stage 9 gynoecium (mock) (S) and after 48 hrs BAP treatment (T), and in a *spt-2* stage 9 gynoecium (mock) (U) and after 48 hrs BAP treatment (V). **(W)** Expression analysis by qRT-PCR of *PIN3* in dissected gynoecia from *spt-12* and *35S*::*SPT* versus wild-type. Error bars represent the SD based on three biological replicates. *P < 0.05, **P = 0.08 (qRT-PCR: ANOVA). **(X)** Localization of *PIN1*::*PIN1-GFP* in the ectopic outgrowths of a gynoecium after five days of BAP treatment. Scale bars: 10 μm (A-C, E-G, I-K, M, N, P, Q), 20 μm (D, H, L, O, R, S-V, X).
(TIF)

S10 Fig PIN3 is necessary for a cytokinin response and with PIN7 for correct gynoecium development. **(A)** Scanning electron microscopy image of a *pin3-4* mutant gynoecium. (**B-D)** Five days BAP-treated gynoecia phenotypes (photos were taken 3–4 weeks after BAP treatment) of wild-type Col-0 with the typical overgrowth of tissue from the repla **(B)**, *pin3-4* lacking the overgrowth of tissue from the repla in 78.2% of the cases **(C)**, and *pin3-4* with a slight phenotype in 21.8% of the cases (n = 330) **(D)**. (**E-H)** Observed gynoecia phenotypes in the *pin3 pin7* double mutant (non-treated plants; n = 277). Phenotypes: 9.3% of the cases the size of the carpels is unequal; 15.2% only one carpel present; 42.2% stem-like structure; 33.3% fused gynoecia-like structures. Insets show a transverse section at the middle of the `ovary`structure. Scale bars: 100 μm (A, E-H), 10 mm (B-D).
(TIF)

S11 Fig TCS signal in cytokinin treated *35S*::*SPT* x *TCS*::*GFP* gynoecia. Expression of the cytokinin response reporter *TCS*::*GFP* in transverse sections of gynoecia at stage 8 and 9 of *35S*::*SPT* **(A, B)**, and *35S*::*SPT* after 48 hours of BAP treatment **(C, D)**. Scale bars: 10 μm.
(TIF)

S12 Fig Protein-protein interaction assays of SPT with ARR proteins. **(A)** Yeast two-hybrid assay with SPT fused to the GAL4 DNA binding domain in combination with itself (homo-dimerization detection) and with 9 type-B ARR proteins (ARR1, ARR2, ARR10, ARR11, ARR12, ARR14, ARR18, ARR20, and ARR21), and also we performed the assay with 8 type-A ARR proteins (ARR3, ARR4, ARR5, ARR6, ARR8, ARR9, ARR15, and ARR16), all fused to the GAL4 activation domain. Positive control reaction: NO TRANSMITTING TRACT (NTT) fused to the GAL4 DNA binding domain in combination with itself (homo-dimerization detection), and NTT against SPT as a negative control. No interaction is observed between SPT and any tested ARR proteins. (**B)** Bimolecular fluorescence complementation (BiFC) assay of SPT with ARR1 in *N*. *tabacum* leaves, where no interaction (no fluorescence) is detected. Positive control for the BiFC assay is SUPPRESSOR OF OVEREXPRESSION OF CO 1 (SOC1) with FRUITFULL (FUL).
(TIF)

## References

[1] Reyes-Olalde JI, Zuñiga-Mayo VM, Chavez Montes RA, Marsch-Martinez N, de Folter S (2013) Inside the gynoecium: at the carpel margin. Trends Plant Sci 18: 644–655. 10.1016/j.tplants.2013.08.002 24008116

[2] Alvarez-Buylla ER, Benitez M, Corvera-Poire A, Chaos Cador A, de Folter S, et al (2010) Flower development. Arabidopsis Book 8: e0127 10.1199/tab.0127 22303253 PMC3244948

[3] Bowman JL, Baum SF, Eshed Y, Putterill J, Alvarez J (1999) Molecular genetics of gynoecium development in Arabidopsis. Curr Top Dev Biol 45: 155–205. 10332605 10.1016/s0070-2153(08)60316-6

[4] Chávez Montes RA, Herrera-Ubaldo H, Serwatowska J, de Folter S (2015) Towards a comprehensive and dynamic gynoecium gene regulatory network. Current Plant Biology 3–4: 3–12.

[5] Sehra B, Franks RG (2015) Auxin and cytokinin act during gynoecial patterning and the development of ovules from the meristematic medial domain. Wiley Interdiscip Rev Dev Biol. 10.1002/wdev.193 25951007

[6] Marsch-Martinez N, de Folter S (2016) Hormonal control of the development of the gynoecium. Curr Opin Plant Biol 29: 104–114. 10.1016/j.pbi.2015.12.006 26799132

[7] Marsch-Martinez N, Ramos-Cruz D, Irepan Reyes-Olalde J, Lozano-Sotomayor P, Zuñiga-Mayo VM, et al (2012) The role of cytokinin during Arabidopsis gynoecia and fruit morphogenesis and patterning. Plant J 72: 222–234. 10.1111/j.1365-313X.2012.05062.x 22640521

[8] Skoog F, Miller CO (1957) Chemical regulation of growth and organ formation in plant tissues cultured in vitro. Symp Soc Exp Biol 11: 118–130. 13486467

[9] Zhao Z, Andersen SU, Ljung K, Dolezal K, Miotk A, et al (2010) Hormonal control of the shoot stem-cell niche. Nature 465: 1089–1092. 10.1038/nature09126 20577215

[10] Ashikari M, Sakakibara H, Lin S, Yamamoto T, Takashi T, et al (2005) Cytokinin oxidase regulates rice grain production. Science 309: 741–745. 10.1126/science.1113373 15976269

[11] Bartrina I, Otto E, Strnad M, Werner T, Schmulling T (2011) Cytokinin regulates the activity of reproductive meristems, flower organ size, ovule formation, and thus seed yield in Arabidopsis thaliana. Plant Cell 23: 69–80. 10.1105/tpc.110.079079 21224426 PMC3051259

[12] Hwang I, Sheen J, Muller B (2012) Cytokinin signaling networks. Annu Rev Plant Biol 63: 353–380. 10.1146/annurev-arplant-042811-105503 22554243

[13] Schaller GE, Bishopp A, Kieber JJ (2015) The Yin-Yang of Hormones: Cytokinin and Auxin Interactions in Plant Development. Plant Cell 27: 44–63. 10.1105/tpc.114.133595 25604447 PMC4330578

[14] Kieber JJ, Schaller GE (2010) The perception of cytokinin: a story 50 years in the making. Plant Physiol 154: 487–492. 10.1104/pp.110.161596 20921170 PMC2948997

[15] Long JA, Moan EI, Medford JI, Barton MK (1996) A member of the KNOTTED class of homeodomain proteins encoded by the STM gene of Arabidopsis. Nature 379: 66–69. 10.1038/379066a0 8538741

[16] Jasinski S, Piazza P, Craft J, Hay A, Woolley L, et al (2005) KNOX action in Arabidopsis is mediated by coordinate regulation of cytokinin and gibberellin activities. Curr Biol 15: 1560–1565. 10.1016/j.cub.2005.07.023 16139211

[17] Yanai O, Shani E, Dolezal K, Tarkowski P, Sablowski R, et al (2005) Arabidopsis KNOXI proteins activate cytokinin biosynthesis. Curr Biol 15: 1566–1571. 10.1016/j.cub.2005.07.060 16139212

[18] Scofield S, Dewitte W, Nieuwland J, Murray JA (2013) The Arabidopsis homeobox gene SHOOT MERISTEMLESS has cellular and meristem-organisational roles with differential requirements for cytokinin and CYCD3 activity. Plant J 75: 53–66. 10.1111/tpj.12198 23573875

[19] Gordon SP, Chickarmane VS, Ohno C, Meyerowitz EM (2009) Multiple feedback loops through cytokinin signaling control stem cell number within the Arabidopsis shoot meristem. Proc Natl Acad Sci U S A 106: 16529–16534. 10.1073/pnas.0908122106 19717465 PMC2752578

[20] Chickarmane VS, Gordon SP, Tarr PT, Heisler MG, Meyerowitz EM (2012) Cytokinin signaling as a positional cue for patterning the apical-basal axis of the growing Arabidopsis shoot meristem. Proc Natl Acad Sci U S A 109: 4002–4007. 10.1073/pnas.1200636109 22345559 PMC3309735

[21] Leibfried A, To JP, Busch W, Stehling S, Kehle A, et al (2005) WUSCHEL controls meristem function by direct regulation of cytokinin-inducible response regulators. Nature 438: 1172–1175. 10.1038/nature04270 16372013

[22] Lenhard M, Jurgens G, Laux T (2002) The WUSCHEL and SHOOTMERISTEMLESS genes fulfil complementary roles in Arabidopsis shoot meristem regulation. Development 129: 3195–3206. 12070094 10.1242/dev.129.13.3195

[23] Gallois JL, Woodward C, Reddy GV, Sablowski R (2002) Combined SHOOT MERISTEMLESS and WUSCHEL trigger ectopic organogenesis in Arabidopsis. Development 129: 3207–3217. 12070095 10.1242/dev.129.13.3207

[24] Werner T, Motyka V, Laucou V, Smets R, Van Onckelen H, et al (2003) Cytokinin-deficient transgenic Arabidopsis plants show multiple developmental alterations indicating opposite functions of cytokinins in the regulation of shoot and root meristem activity. Plant Cell 15: 2532–2550. 10.1105/tpc.014928 14555694 PMC280559

[25] Larsson E, Franks RG, Sundberg E (2013) Auxin and the Arabidopsis thaliana gynoecium. J Exp Bot 64: 2619–2627. 10.1093/jxb/ert099 23585670

[26] Weijers D, Wagner D (2016) Transcriptional responses to the auxin hormone. Annu Rev Plant Biol 67: 21.21–21.36. 10.1146/annurev-arplant-043015-112122 26905654

[27] Robert HS, Crhak Khaitova L, Mroue S, Benkova E (2015) The importance of localized auxin production for morphogenesis of reproductive organs and embryos in Arabidopsis. J Exp Bot 66: 5029–5042. 10.1093/jxb/erv256 26019252

[28] Nemhauser JL, Feldman LJ, Zambryski PC (2000) Auxin and ETTIN in Arabidopsis gynoecium morphogenesis. Development 127: 3877–3888. 10952886 10.1242/dev.127.18.3877

[29] Kuusk S, Sohlberg JJ, Magnus Eklund D, Sundberg E (2006) Functionally redundant SHI family genes regulate Arabidopsis gynoecium development in a dose-dependent manner. Plant J 47: 99–111. 10.1111/j.1365-313X.2006.02774.x 16740146

[30] Sohlberg JJ, Myrenas M, Kuusk S, Lagercrantz U, Kowalczyk M, et al (2006) STY1 regulates auxin homeostasis and affects apical-basal patterning of the Arabidopsis gynoecium. Plant J 47: 112–123. 10.1111/j.1365-313X.2006.02775.x 16740145

[31] Staldal V, Sohlberg JJ, Eklund DM, Ljung K, Sundberg E (2008) Auxin can act independently of CRC, LUG, SEU, SPT and STY1 in style development but not apical-basal patterning of the Arabidopsis gynoecium. New Phytol 180: 798–808. 10.1111/j.1469-8137.2008.02625.x 18811619

[32] Zuñiga-Mayo VM, Reyes-Olalde JI, Marsch-Martinez N, de Folter S (2014) Cytokinin treatments affect the apical-basal patterning of the Arabidopsis gynoecium and resemble the effects of polar auxin transport inhibition. Front Plant Sci 5: 191 10.3389/fpls.2014.00191 24860582 PMC4030163

[33] van Gelderen K, van Rongen M, Liu A, Otten A, Offringa R (2016) An INDEHISCENT-controlled auxin response specifies the separation layer in early Arabidopsis fruit. Molecular Plant 9: 857–869. 10.1016/j.molp.2016.03.005 26995296

[34] Ripoll JJ, Bailey LJ, Mai Q-A, Wu SL, Hon CT, et al (2015) microRNA regulation of fruit growth. Nature Plants 1: 15036 10.1038/nplants.2015.36 27247036

[35] Larsson E, Roberts CJ, Claes AR, Franks RG, Sundberg E (2014) Polar auxin transport is essential for medial versus lateral tissue specification and vascular-mediated valve outgrowth in Arabidopsis gynoecia. Plant Physiol 166: 1998–2012. 10.1104/pp.114.245951 25332506 PMC4256862

[36] Nole-Wilson S, Azhakanandam S, Franks RG (2010) Polar auxin transport together with aintegumenta and revoluta coordinate early Arabidopsis gynoecium development. Dev Biol 346: 181–195. 10.1016/j.ydbio.2010.07.016 20654611

[37] de Folter S (2016) Auxin Is Required for Valve Margin Patterning in Arabidopsis After All. Mol Plant 9: 768–770. 10.1016/j.molp.2016.05.005 27212385

[38] Moubayidin L, Ostergaard L (2014) Dynamic control of auxin distribution imposes a bilateral-to-radial symmetry switch during gynoecium development. Curr Biol 24: 2743–2748. 10.1016/j.cub.2014.09.080 25455035 PMC4245708

[39] Girin T, Paicu T, Stephenson P, Fuentes S, Korner E, et al (2011) INDEHISCENT and SPATULA interact to specify carpel and valve margin tissue and thus promote seed dispersal in Arabidopsis. Plant Cell 23: 3641–3653. 10.1105/tpc.111.090944 21990939 PMC3229140

[40] Dello Ioio R, Nakamura K, Moubayidin L, Perilli S, Taniguchi M, et al (2008) A genetic framework for the control of cell division and differentiation in the root meristem. Science 322: 1380–1384. 10.1126/science.1164147 19039136

[41] Bishopp A, Help H, El-Showk S, Weijers D, Scheres B, et al (2011) A mutually inhibitory interaction between auxin and cytokinin specifies vascular pattern in roots. Curr Biol 21: 917–926. 10.1016/j.cub.2011.04.017 21620702

[42] De Rybel B, Adibi M, Breda AS, Wendrich JR, Smit ME, et al (2014) Plant development. Integration of growth and patterning during vascular tissue formation in Arabidopsis. Science 345: 1255215 25104393 10.1126/science.1255215

[43] Pernisova M, Klima P, Horak J, Valkova M, Malbeck J, et al (2009) Cytokinins modulate auxin-induced organogenesis in plants via regulation of the auxin efflux. Proc Natl Acad Sci U S A 106: 3609–3614. 10.1073/pnas.0811539106 19211794 PMC2640219

[44] Cheng ZJ, Wang L, Sun W, Zhang Y, Zhou C, et al (2013) Pattern of auxin and cytokinin responses for shoot meristem induction results from the regulation of cytokinin biosynthesis by AUXIN RESPONSE FACTOR3. Plant Physiol 161: 240–251. 10.1104/pp.112.203166 23124326 PMC3532255

[45] Alvarez J, Smyth DR (1999) CRABS CLAW and SPATULA, two Arabidopsis genes that control carpel development in parallel with AGAMOUS. Development 126: 2377–2386. 10225997 10.1242/dev.126.11.2377

[46] Alvarez J, Smyth DR (2002) CRABS CLAW and SPATULA genes regulate growth and pattern formation during gynoecium development in Arabidopsis thaliana. International journal of plant sciences 163: 17–41.

[47] Heisler MG, Atkinson A, Bylstra YH, Walsh R, Smyth DR (2001) SPATULA, a gene that controls development of carpel margin tissues in Arabidopsis, encodes a bHLH protein. Development 128: 1089–1098. 11245574 10.1242/dev.128.7.1089

[48] Groszmann M, Bylstra Y, Lampugnani ER, Smyth DR (2010) Regulation of tissue-specific expression of SPATULA, a bHLH gene involved in carpel development, seedling germination, and lateral organ growth in Arabidopsis. J Exp Bot 61: 1495–1508. 10.1093/jxb/erq015 20176890 PMC2837263

[49] Smyth DR, Bowman JL, Meyerowitz EM (1990) Early flower development in Arabidopsis. Plant Cell 2: 755–767. 10.1105/tpc.2.8.755 2152125 PMC159928

[50] Muller B, Sheen J (2008) Cytokinin and auxin interaction in root stem-cell specification during early embryogenesis. Nature 453: 1094–1097. 10.1038/nature06943 18463635 PMC2601652

[51] Argyros RD, Mathews DE, Chiang YH, Palmer CM, Thibault DM, et al (2008) Type B response regulators of Arabidopsis play key roles in cytokinin signaling and plant development. Plant Cell 20: 2102–2116. 10.1105/tpc.108.059584 18723577 PMC2553617

[52] Mason MG, Mathews DE, Argyros DA, Maxwell BB, Kieber JJ, et al (2005) Multiple type-B response regulators mediate cytokinin signal transduction in Arabidopsis. Plant Cell 17: 3007–3018. 10.1105/tpc.105.035451 16227453 PMC1276026

[53] Ishida K, Yamashino T, Yokoyama A, Mizuno T (2008) Three type-B response regulators, ARR1, ARR10 and ARR12, play essential but redundant roles in cytokinin signal transduction throughout the life cycle of Arabidopsis thaliana. Plant Cell Physiol 49: 47–57. 10.1093/pcp/pcm165 18037673

[54] Yokoyama A, Yamashino T, Amano Y, Tajima Y, Imamura A, et al (2007) Type-B ARR transcription factors, ARR10 and ARR12, are implicated in cytokinin-mediated regulation of protoxylem differentiation in roots of Arabidopsis thaliana. Plant Cell Physiol 48: 84–96. 10.1093/pcp/pcl040 17132632

[55] Schuster C, Gaillochet C, Lohmann JU (2015) Arabidopsis *HECATE* genes function in phytohormone control during gynoecium development. Development 142: 3343–3350. 10.1242/dev.120444 26293302 PMC4631749

[56] Toledo-Ortiz G, Huq E, Quail PH (2003) The Arabidopsis basic/helix-loop-helix transcription factor family. Plant Cell 15: 1749–1770. 10.1105/tpc.013839 12897250 PMC167167

[57] Reymond MC, Brunoud G, Chauvet A, Martinez-Garcia JF, Martin-Magniette ML, et al (2012) A light-regulated genetic module was recruited to carpel development in Arabidopsis following a structural change to SPATULA. Plant Cell 24: 2812–2825. 10.1105/tpc.112.097915 22851763 PMC3426116

[58] Ballester P, Navarrete-Gomez M, Carbonero P, Onate-Sanchez L, Ferrandiz C (2015) Leaf expansion in Arabidopsis is controlled by a TCP-NGA regulatory module likely conserved in distantly related species. Physiologia Plantarum 155: 21–32. 10.1111/ppl.12327 25625546

[59] Hellens RP, Allan AC, Friel EN, Bolitho K, Grafton K, et al (2005) Transient expression vectors for functional genomics, quantification of promoter activity and RNA silencing in plants. Plant Methods 1: 13 10.1186/1746-4811-1-13 16359558 PMC1334188

[60] Makkena S, Lamb RS (2013) The bHLH transcription factor SPATULA regulates root growth by controlling the size of the root meristem. BMC Plant Biol 13: 1 10.1186/1471-2229-13-1 23280064 PMC3583232

[61] Stepanova AN, Robertson-Hoyt J, Yun J, Benavente LM, Xie DY, et al (2008) TAA1-mediated auxin biosynthesis is essential for hormone crosstalk and plant development. Cell 133: 177–191. 10.1016/j.cell.2008.01.047 18394997

[62] Bhargava A, Clabaugh I, To JP, Maxwell BB, Chiang YH, et al (2013) Identification of cytokinin-responsive genes using microarray meta-analysis and RNA-Seq in Arabidopsis. Plant Physiol 162: 272–294. 10.1104/pp.113.217026 23524861 PMC3641208

[63] Sakai H, Aoyama T, Oka A (2000) Arabidopsis ARR1 and ARR2 response regulators operate as transcriptional activators. Plant J 24: 703–711. 11135105 10.1046/j.1365-313x.2000.00909.x

[64] Sakai H, Honma T, Aoyama T, Sato S, Kato T, et al (2001) ARR1, a transcription factor for genes immediately responsive to cytokinins. Science 294: 1519–1521. 10.1126/science.1065201 11691951

[65] Moubayidin L, Di Mambro R, Sozzani R, Pacifici E, Salvi E, et al (2013) Spatial coordination between stem cell activity and cell differentiation in the root meristem. Dev Cell 26: 405–415. 10.1016/j.devcel.2013.06.025 23987513 PMC4839959

[66] Benkova E, Michniewicz M, Sauer M, Teichmann T, Seifertova D, et al (2003) Local, efflux-dependent auxin gradients as a common module for plant organ formation. Cell 115: 591–602. 14651850 10.1016/s0092-8674(03)00924-3

[67] Okada K, Ueda J, Komaki MK, Bell CJ, Shimura Y (1991) Requirement of the Auxin Polar Transport System in Early Stages of Arabidopsis Floral Bud Formation. Plant Cell 3: 677–684. 10.1105/tpc.3.7.677 12324609 PMC160035

[68] Blilou I, Xu J, Wildwater M, Willemsen V, Paponov I, et al (2005) The PIN auxin efflux facilitator network controls growth and patterning in Arabidopsis roots. Nature 433: 39–44. 10.1038/nature03184 15635403

[69] Mahonen AP, Bishopp A, Higuchi M, Nieminen KM, Kinoshita K, et al (2006) Cytokinin signaling and its inhibitor AHP6 regulate cell fate during vascular development. Science 311: 94–98. 10.1126/science.1118875 16400151

[70] Besnard F, Refahi Y, Morin V, Marteaux B, Brunoud G, et al (2014) Cytokinin signalling inhibitory fields provide robustness to phyllotaxis. Nature 505: 417–421. 10.1038/nature12791 24336201

[71] Longabaugh WJ, Davidson EH, Bolouri H (2005) Computational representation of developmental genetic regulatory networks. Dev Biol 283: 1–16. 10.1016/j.ydbio.2005.04.023 15907831

[72] Faure E, Peter IS, Davidson EH (2013) A new software package for predictive gene regulatory network modeling and redesign. J Comput Biol 20: 419–423. 10.1089/cmb.2012.0297 23614576 PMC3667423

[73] Peter IS, Davidson EH (2015) Genomic Control Process: Development and Evolution. San Diago, CA, USA: Academic Press.

[74] Mangan S, Alon U (2003) Structure and function of the feed-forward loop network motif. Proc Natl Acad Sci U S A 100: 11980–11985. 10.1073/pnas.2133841100 14530388 PMC218699

[75] Chen Q, Liu Y, Maere S, Lee E, Van Isterdael G, et al (2015) A coherent transcriptional feed-forward motif model for mediating auxin-sensitive PIN3 expression during lateral root development. Nat Commun 6: 8821 10.1038/ncomms9821 26578065 PMC4673502

[76] Qiu K, Li Z, Yang Z, Chen J, Wu S, et al (2015) EIN3 and ORE1 Accelerate Degreening during Ethylene-Mediated Leaf Senescence by Directly Activating Chlorophyll Catabolic Genes in Arabidopsis. PLoS Genet 11: e1005399 10.1371/journal.pgen.1005399 26218222 PMC4517869

[77] Seaton DD, Smith RW, Song YH, MacGregor DR, Stewart K, et al (2015) Linked circadian outputs control elongation growth and flowering in response to photoperiod and temperature. Mol Syst Biol 11: 776 10.15252/msb.20145766 25600997 PMC4332151

[78] Roeder AH, Yanofsky MF (2006) Fruit development in Arabidopsis. Arabidopsis Book 4: e0075 10.1199/tab.0075 22303227 PMC3243326

[79] Ferrandiz C, Fourquin C, Prunet N, Scutt CP, Sundberg E, et al (2010) Carpel Development Advances in Botanical Research, Vol 55 55: 1–73.

[80] Martínez-Laborda A, Vera A (2009) Arabidopsis Fruit Development In: Østergaard L, editor. Annual Plant Reviews Volume 38: Fruit Development and Seed Dispersal. Oxford, UK: Wiley-Blackwell pp. 172–203.

[81] Marsch-Martínez N, Reyes-Olalde JI, Ramos-Cruz D, Lozano-Sotomayor P, Zúñiga-Mayo VM, et al (2012) Hormones talking: does hormonal cross-talk shape the Arabidopsis gynoecium? Plant Signal Behav 7: 1698–1701. 10.4161/psb.22422 23072997 PMC3578912

[82] Balanza V, Navarrete M, Trigueros M, Ferrandiz C (2006) Patterning the female side of Arabidopsis: the importance of hormones. J Exp Bot 57: 3457–3469. 10.1093/jxb/erl188 17023565

[83] Kamiuchi Y, Yamamoto K, Furutani M, Tasaka M, Aida M (2014) The CUC1 and CUC2 genes promote carpel margin meristem formation during Arabidopsis gynoecium development. Front Plant Sci 5: 165 10.3389/fpls.2014.00165 24817871 PMC4012194

[84] Scofield S, Dewitte W, Murray JA (2007) The KNOX gene SHOOT MERISTEMLESS is required for the development of reproductive meristematic tissues in Arabidopsis. Plant J 50: 767–781. 10.1111/j.1365-313X.2007.03095.x 17461793

[85] Li K, Yu R, Fan LM, Wei N, Chen H, et al (2016) DELLA-mediated PIF degradation contributes to coordination of light and gibberellin signalling in Arabidopsis. Nat Commun 7: 11868 10.1038/ncomms11868 27282989 PMC4906400

[86] Oh E, Zhu JY, Bai MY, Arenhart RA, Sun Y, et al (2014) Cell elongation is regulated through a central circuit of interacting transcription factors in the Arabidopsis hypocotyl. Elife 3. 10.7554/eLife.03031 PMC4075450 24867218

[87] Oh E, Zhu JY, Wang ZY (2012) Interaction between BZR1 and PIF4 integrates brassinosteroid and environmental responses. Nat Cell Biol 14: 802–809. 10.1038/ncb2545 22820378 PMC3703456

[88] Sharma N, Xin R, Kim DH, Sung S, Lange T, et al (2016) NO FLOWERING IN SHORT DAY (NFL) is a bHLH transcription factor that promotes flowering specifically under short-day conditions in Arabidopsis. Development 143: 682–690. 10.1242/dev.128595 26758694 PMC4760320

[89] Varaud E, Brioudes F, Szecsi J, Leroux J, Brown S, et al (2011) AUXIN RESPONSE FACTOR8 regulates Arabidopsis petal growth by interacting with the bHLH transcription factor BIGPETALp. Plant Cell 23: 973–983. 10.1105/tpc.110.081653 21421811 PMC3082276

[90] Savaldi-Goldstein S, Chory J (2008) Growth coordination and the shoot epidermis. Curr Opin Plant Biol 11: 42–48. 10.1016/j.pbi.2007.10.009 18065257 PMC2274781

[91] Schuster C, Gaillochet C, Medzihradszky A, Busch W, Daum G, et al (2014) A regulatory framework for shoot stem cell control integrating metabolic, transcriptional, and phytohormone signals. Dev Cell 28: 438–449. 10.1016/j.devcel.2014.01.013 24576426

[92] Gremski K, Ditta G, Yanofsky MF (2007) The HECATE genes regulate female reproductive tract development in Arabidopsis thaliana. Development 134: 3593–3601. 10.1242/dev.011510 17855426

[93] Lucero LE, Uberti-Manassero NG, Arce AL, Colombatti F, Alemano SG, et al (2015) TCP15 modulates cytokinin and auxin responses during gynoecium development in Arabidopsis. Plant J 84: 267–282. 10.1111/tpj.12992 26303297

[94] Nibau C, Di Stilio VS, Wu HM, Cheung AY (2011) Arabidopsis and Tobacco superman regulate hormone signalling and mediate cell proliferation and differentiation. J Exp Bot 62: 949–961. 10.1093/jxb/erq325 20980362 PMC3022392

[95] Eshed Y, Baum SF, Bowman JL (1999) Distinct mechanisms promote polarity establishment in carpels of Arabidopsis. Cell 99: 199–209. 10535738 10.1016/s0092-8674(00)81651-7

[96] Sessions RA, Zambryski PC (1995) Arabidopsis gynoecium structure in the wild and in ettin mutants. Development 121: 1519–1532. 7789281 10.1242/dev.121.5.1519

[97] Otsuga D, DeGuzman B, Prigge MJ, Drews GN, Clark SE (2001) REVOLUTA regulates meristem initiation at lateral positions. Plant J 25: 223–236. 11169198 10.1046/j.1365-313x.2001.00959.x

[98] Eshed Y, Baum SF, Perea JV, Bowman JL (2001) Establishment of polarity in lateral organs of plants. Curr Biol 11: 1251–1260. 11525739 10.1016/s0960-9822(01)00392-x

[99] Kerstetter RA, Bollman K, Taylor RA, Bomblies K, Poethig RS (2001) KANADI regulates organ polarity in Arabidopsis. Nature 411: 706–709. 10.1038/35079629 11395775

[100] Bowman JL, Floyd SK (2008) Patterning and polarity in seed plant shoots. Annu Rev Plant Biol 59: 67–88. 10.1146/annurev.arplant.57.032905.105356 18031217

[101] Pires HR, Monfared MM, Shemyakina EA, Fletcher JC (2014) ULTRAPETALA trxG genes interact with KANADI transcription factor genes to regulate Arabidopsis gynoecium patterning. Plant Cell 26: 4345–4361. 10.1105/tpc.114.131250 25381352 PMC4277222

[102] Endress PK, Igersheim A (2000) Gynoecium structure and evolution in basal angiosperms. Int J Pl Sci 161.

[103] Doyle JA (2012) Phylogenetic Analyses and Morphological Innovations in Land Plants In: Ambrose BA, Purugganan M, editors. Annual Plant Reviews Volume 45: The Evolution of Plant Form. Chichester, West Sussex, UK: John Wiley & Sons, Ltd pp. 1–50.

[104] Pabon-Mora N, Wong GK, Ambrose BA (2014) Evolution of fruit development genes in flowering plants. Front Plant Sci 5: 300 10.3389/fpls.2014.00300 25018763 PMC4071287

[105] Pils B, Heyl A (2009) Unraveling the evolution of cytokinin signaling. Plant Physiol 151: 782–791. 10.1104/pp.109.139188 19675156 PMC2754637

[106] Bennett T, Brockington SF, Rothfels C, Graham SW, Stevenson D, et al (2014) Paralogous radiations of PIN proteins with multiple origins of noncanonical PIN structure. Mol Biol Evol 31: 2042–2060. 10.1093/molbev/msu147 24758777 PMC4104312

[107] Tivendale ND, Ross JJ, Cohen JD (2014) The shifting paradigms of auxin biosynthesis. Trends Plant Sci 19: 44–51. 24524164 10.1016/j.tplants.2013.09.012

[108] Pfannebecker KC, Lange M, Rupp O, Becker A (2017) Seed plant specific gene lineages involved in carpel development. Mol Biol Evol In press. 10.1093/molbev/msw297 28087776

[109] Livak KJ, Schmittgen TD (2001) Analysis of relative gene expression data using real-time quantitative PCR and the 2(-Delta Delta C(T)) Method. Methods 25: 402–408. 10.1006/meth.2001.1262 11846609

[110] Gonzalez-Reig S, Ripoll JJ, Vera A, Yanofsky MF, Martinez-Laborda A (2012) Antagonistic gene activities determine the formation of pattern elements along the mediolateral axis of the Arabidopsis fruit. PLoS Genet 8: e1003020 10.1371/journal.pgen.1003020 23133401 PMC3486860

[111] Matias-Hernandez L, Battaglia R, Galbiati F, Rubes M, Eichenberger C, et al (2010) VERDANDI is a direct target of the MADS domain ovule identity complex and affects embryo sac differentiation in Arabidopsis. Plant Cell 22: 1702–1715. 10.1105/tpc.109.068627 20581305 PMC2910977

[112] Penfield S, Josse EM, Kannangara R, Gilday AD, Halliday KJ, et al (2005) Cold and light control seed germination through the bHLH transcription factor SPATULA. Curr Biol 15: 1998–2006. 10.1016/j.cub.2005.11.010 16303558

[113] Earley KW, Haag JR, Pontes O, Opper K, Juehne T, et al (2006) Gateway-compatible vectors for plant functional genomics and proteomics. Plant J 45: 616–629. 10.1111/j.1365-313X.2005.02617.x 16441352

[114] Marín-de la Rosa N, Pfeiffer A, Hill K, Locascio A, Bhalerao RP, et al (2015) Genome Wide Binding Site Analysis Reveals Transcriptional Coactivation of Cytokinin-Responsive Genes by DELLA Proteins. PLoS Genet 11: e1005337 10.1371/journal.pgen.1005337 26134422 PMC4489807

[115] Espley RV, Brendolise C, Chagne D, Kutty-Amma S, Green S, et al (2009) Multiple repeats of a promoter segment causes transcription factor autoregulation in red apples. Plant Cell 21: 168–183. 10.1105/tpc.108.059329 19151225 PMC2648084

[116] Reyes-Olalde JI, Marsch-Martinez N, de Folter S (2015) Imaging early stages of the female reproductive structure of Arabidopsis by confocal laser scanning microscopy. Dev Dyn 244: 1286–1290. 10.1002/dvdy.24301 26149964

[117] Marsch-Martinez N, Zuñiga-Mayo VM, Herrera-Ubaldo H, Ouwerkerk PB, Pablo-Villa J, et al (2014) The NTT transcription factor promotes replum development in Arabidopsis fruits. Plant J 80: 69–81. 10.1111/tpj.12617 25039392

[118] Zuniga-Mayo VM, Marsch-Martinez N, de Folter S (2012) JAIBA, a class-II HD-ZIP transcription factor involved in the regulation of meristematic activity, and important for correct gynoecium and fruit development in Arabidopsis. Plant J 71: 314–326. 10.1111/j.1365-313X.2012.04990.x 22409594

[119] Dortay H, Mehnert N, Burkle L, Schmulling T, Heyl A (2006) Analysis of protein interactions within the cytokinin-signaling pathway of Arabidopsis thaliana. FEBS J 273: 4631–4644. 10.1111/j.1742-4658.2006.05467.x 16965536

[120] de Folter S, Immink RG (2011) Yeast protein-protein interaction assays and screens. Methods Mol Biol 754: 145–165. 10.1007/978-1-61779-154-3_8 21720951

[121] Goehler H, Lalowski M, Stelzl U, Waelter S, Stroedicke M, et al (2004) A protein interaction network links GIT1, an enhancer of huntingtin aggregation, to Huntington's disease. Mol Cell 15: 853–865. 10.1016/j.molcel.2004.09.016 15383276

[122] Belda-Palazon B, Ruiz L, Marti E, Tarraga S, Tiburcio AF, et al (2012) Aminopropyltransferases involved in polyamine biosynthesis localize preferentially in the nucleus of plant cells. PLoS One 7: e46907 10.1371/journal.pone.0046907 23056524 PMC3466176

