# Supplementary material for: The bHLH transcription factor SPATULA enables cytokinin signaling, and both activate auxin biosynthesis and transport genes at the medial domain of the gynoecium
Source: PLoS Genet. 2017 Apr 7;13(4):e1006726. doi: 10.1371/journal.pgen.1006726 (PMC5400277; doi:10.1371/journal.pgen.1006726)
Supplement: S1 Table — (PDF) [file pgen.1006726.s001.pdf]

S1 Table. Primers used in this study.

Gene	Accession	Forward primer	Reverse primer	Purpose	Reference
ARR12	AT2G25180	CACGATGAAGCAGGAACAGA	TTCTGAGTGAACTAAACCCTCCA	qRT-PCR	Muller B, Sheen J. (2008)[1]
ARR1	AT3G16857	GCAAGTCACCTCCAGAAATACC	ATCCTGACCCGTCATAAACG	qRT-PCR	Muller B, Sheen J. (2008)[1]
ARR10	AT4G31920	GACACAGGAACAGAGCCAATC	TATGCATGTTCCGAGTGAGC	qRT-PCR	Muller B, Sheen J. (2008)[1]
ACT2/7	AT1G49240	CGTACAACCGGTATTGTGCTGGAT	GCTTGGTGCAAGTGCTGTGATTTC	qRT-PCR	This study
SPT	AT4G36930	CAGCTCCAAGTTCAGATGTT	GAGCTTGTTCCCCGGTTATG	qRT-PCR	Ichihashi *et al*., (2010)[2]
PIN3	AT1G70940	GACCAGGTGATGCCGAATA	CTGATGCTGGTCTTGGAATG	qRT-PCR	Bennett *et al*., (2006)[3]
***PIN3* a**	AT1G70940	AGTCCAAAGATCAGAGTAAACAGAGG	CACGCGTCTTGTCCATAATGAAAG	CHiP	This study
***PIN3* b**	AT1G70940	CACGTGTTTAAGCCACCAGTAGCC	CACGTGAACAAAAGTATACGGTTTAGCC	CHiP	This study
ARR1	AT3G16857	CGATAGATGGAGAGGTCGATGC	CTATTGCGACACGTGTCCACC	ChIP	This study
TAA1	AT1G70560	TCCATTTAAAAAGCAGATACCAGTC	AGAGAATAGTAGGTCGAAGTGT	ChIP	This study

ACTIN2/7	AT1G49240	CCAATCGTGAGAAAATGACTCAG	CCAAACGCAGAATAGCATGTGG	ChIP	Matias-Hernandez *et al.*, (2010)[4]
ARR1	AT3g16857	ATTTAGGTGACACTATAGATTACTT CACGGTGTCCCCACG	TAATACGACTCACTATAGGGGTGTCTTGAC ATGGACGAAGAAGAG	*In situ* hybridization	This study
pPIN3	AT1G70940	CCCGGGAGAGATTATTAAAACATCA ATTAACGTCA	CCATGGCCACGTAGAGAGGA ATCACGGCGGT	LUC assay	This study
pARR1	AT3g16857	CCCGGGAAAGCTTCTCAGCAACGTG ATT	CCATGGCTCTCTCTATGTAGCTCGAA	LUC assay	This study
pTAA1	AT1G70560	GGATAGAGCGACTCTCACGTC	CTTCTTCTTCTTGGTTTGGTCGTTTG	LUC assay	This study
SPT	AT4G36930	ATGATATCACAGAGAGAAGAAA	TCAAGTAATTCGATCTTTTAGG	35S::SPT LUC assay	This study
SPT	AT4G36930	CACCTTTTTTGTTGTTGGTGTAATGA TAT	GGACACTGTTCAAGTAATTCG	Y2H and BiFC	This study

REFERENCES

1 Muller, B. & Sheen, J. Cytokinin and auxin interaction in root stem-cell specification during early embryogenesis. *Nature* **453**, 1094-1097 (2008).

2 Ichihashi, Y., Horiguchi, G., Gleissberg, S. & Tsukaya, H. The bHLH transcription factor SPATULA controls final leaf size in Arabidopsis thaliana. *Plant Cell Physiol* **51**, 252-261 (2010).

3 Bennett, T. *et al.* The Arabidopsis MAX pathway controls shoot branching by regulating auxin transport. *Curr Biol* **16**, 553-563 (2006).

4 Matias-Hernandez, L. *et al.* VERDANDI is a direct target of the MADS domain ovule identity complex and affects embryo sac differentiation in Arabidopsis. *Plant Cell* **22**, 1702-1715 (2010).
