# Supplementary figures and images for: The bHLH transcription factor SPATULA enables cytokinin signaling, and both activate auxin biosynthesis and transport genes at the medial domain of the gynoecium
Source: PLoS Genet. 2017 Apr 7;13(4):e1006726. doi: 10.1371/journal.pgen.1006726 (PMC5400277; doi:10.1371/journal.pgen.1006726)

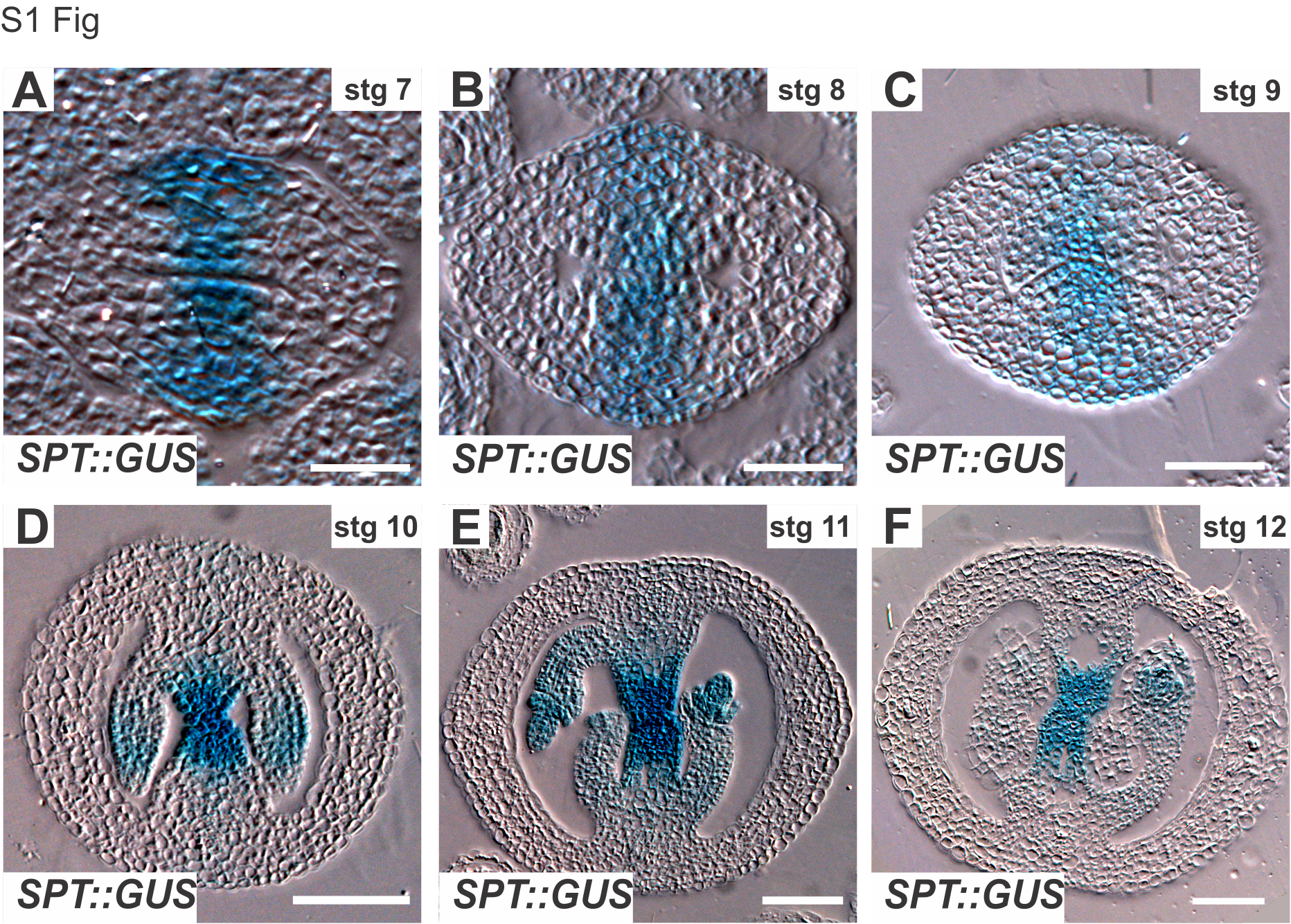

Supplement: S1 Fig — (A-F) Expression of SPT::GUS during gynoecium development at stage 7, 8, 9, 10, 11, and 12, respectively. Scale bars: 20 μm (A-C), 40 μm (D-F). (TIF) [file pgen.1006726.s002.tif]

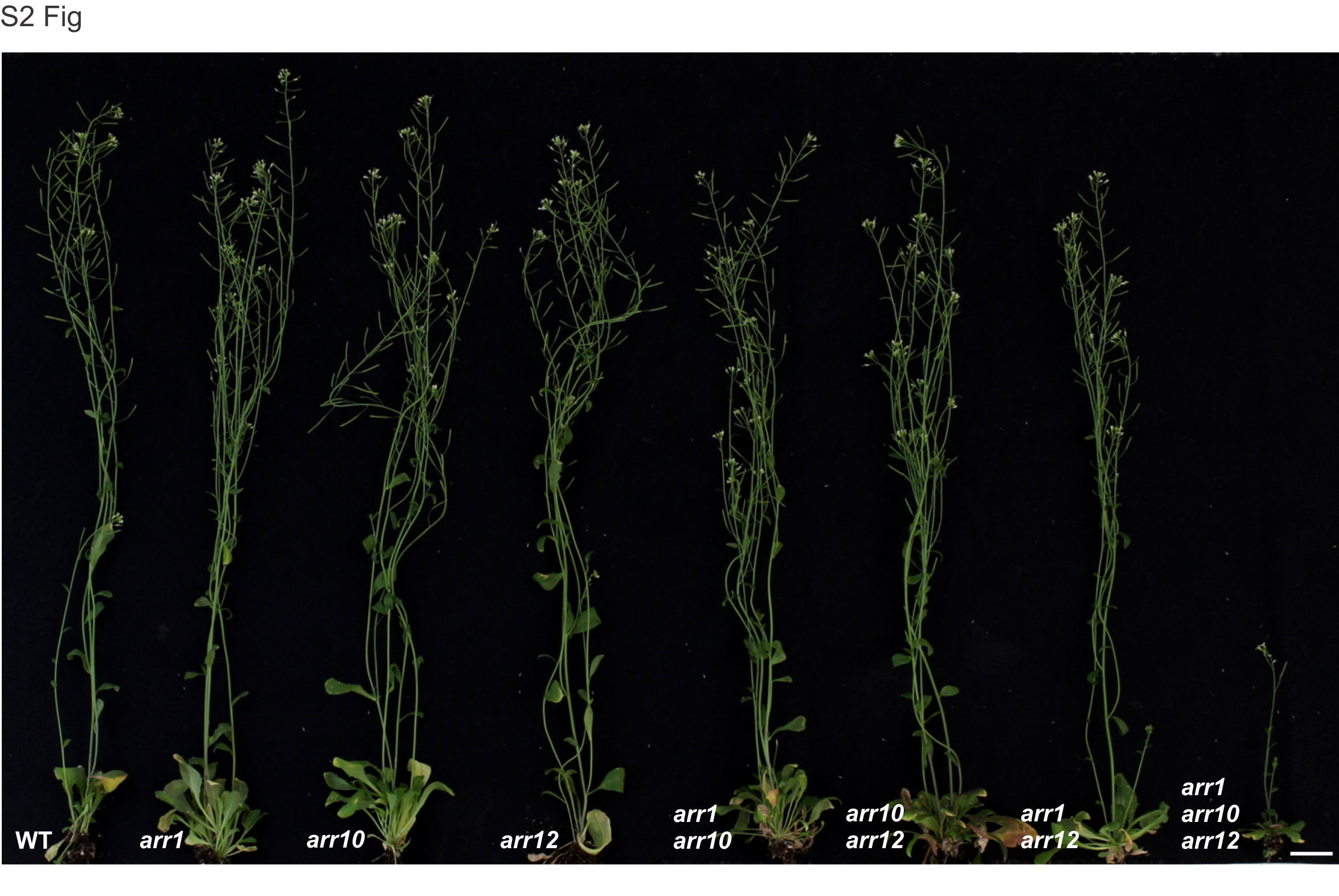

Supplement: S2 Fig — Photos of plants of 73 days old of wild-type (Col-0), arr1, arr10, arr12, arr1 arr10, arr10 arr12, arr1 arr12, and arr1 arr10 arr12. Scale bar: 3 cm. (TIF) [file pgen.1006726.s003.tif]

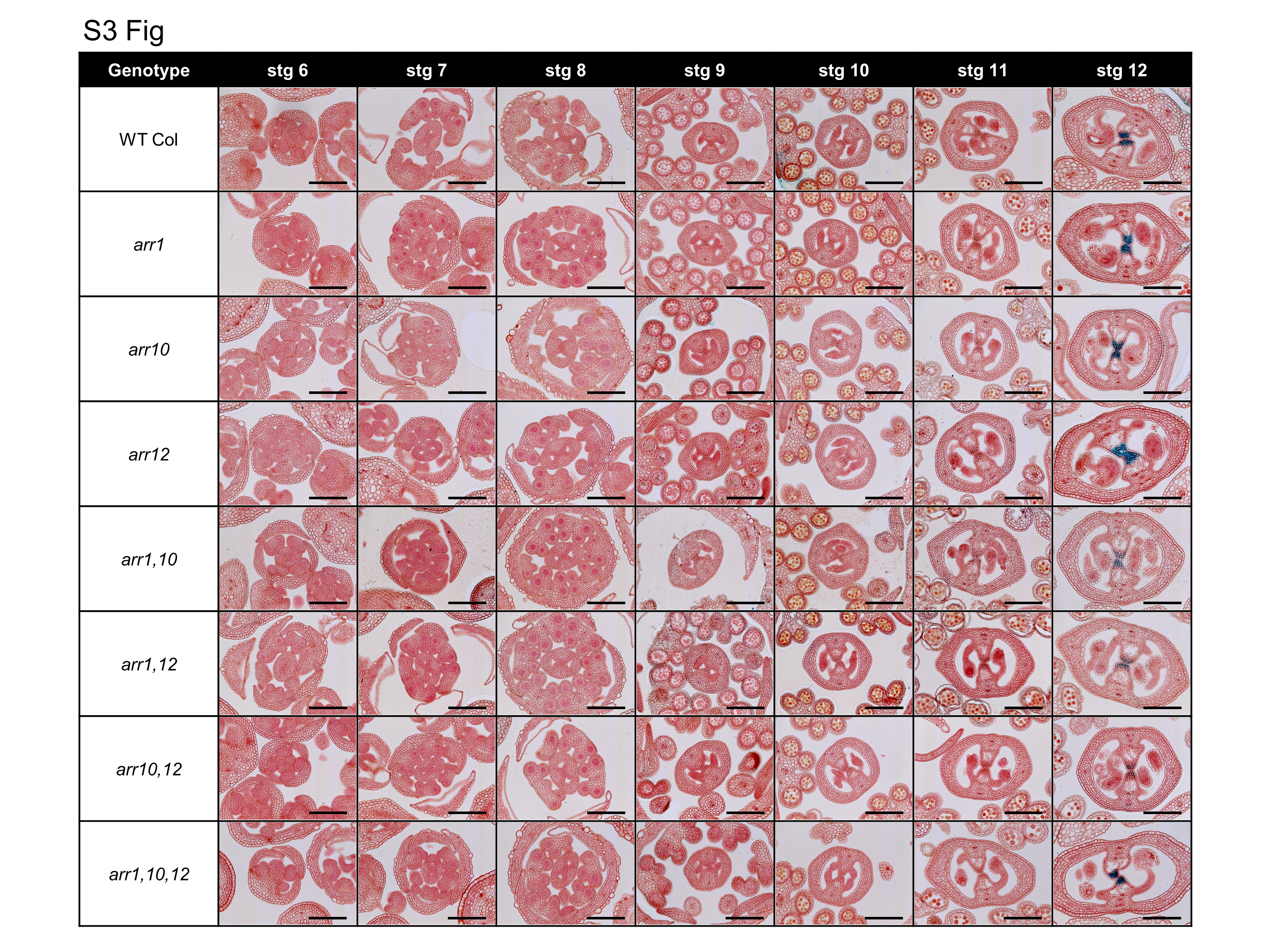

Supplement: S3 Fig — Transverse sections of the ovary region of stage 6–12 gynoecia of wild-type, arr1, arr10, arr12, arr1 arr10, arr10 arr12, arr1 arr12, and arr1 arr10 arr12. The photo of the stage 12 gynoecium of the triple type-B arr mutant is an example of a section with an apparently normal transmitting tract. Scale bars: 100 μm. (TIF) [file pgen.1006726.s004.tif]

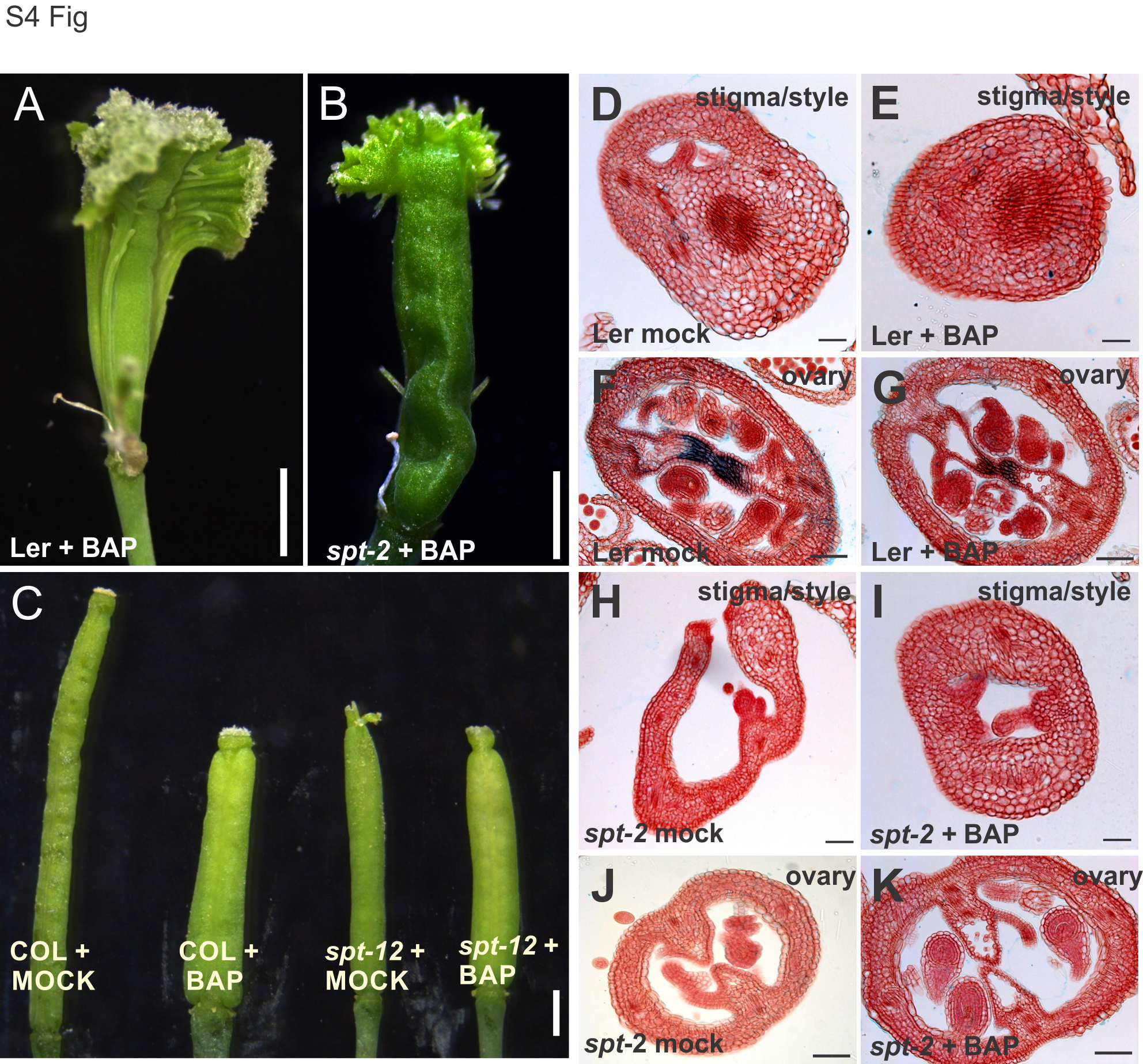

Supplement: S4 Fig — (A, B) Phenotypes of wild-type Ler (A) and spt-2 (B) gynoecia treated with BAP for 5 days. The photos were taken 3–4 weeks after the BAP treatment. In (B) an example is shown of a spt-2 gynoecium presenting a minor effect to BAP in the replum outgrowth phenotype (only in 12.5% of the cases). (C) Phenotypes of wild-type Col-0 (left) and of spt-12 (right) gynoecia treated with mock or BAP for 48 hours. The photos were taken 1 day after the BAP treatment. (D, E, H, I) Transverse sections of stigma/style region of gynoecia of wild-type Ler (mock) (D) and spt-2 (mock) (H), and of 48 hours BAP-treated gynoecia of wild-type Ler (E) and of spt-2 (I). (F, G, J, K) Transverse sections of the ovary region of gynoecia of wild-type Ler (mock) (F) and spt-2 (mock) (J), and of 48 hours BAP-treated gynoecia of wild-type Ler (G) and of spt-2 (K). Scale bars: 10 mm (A-C), 150 μm (D-K). (TIF) [file pgen.1006726.s005.tif]

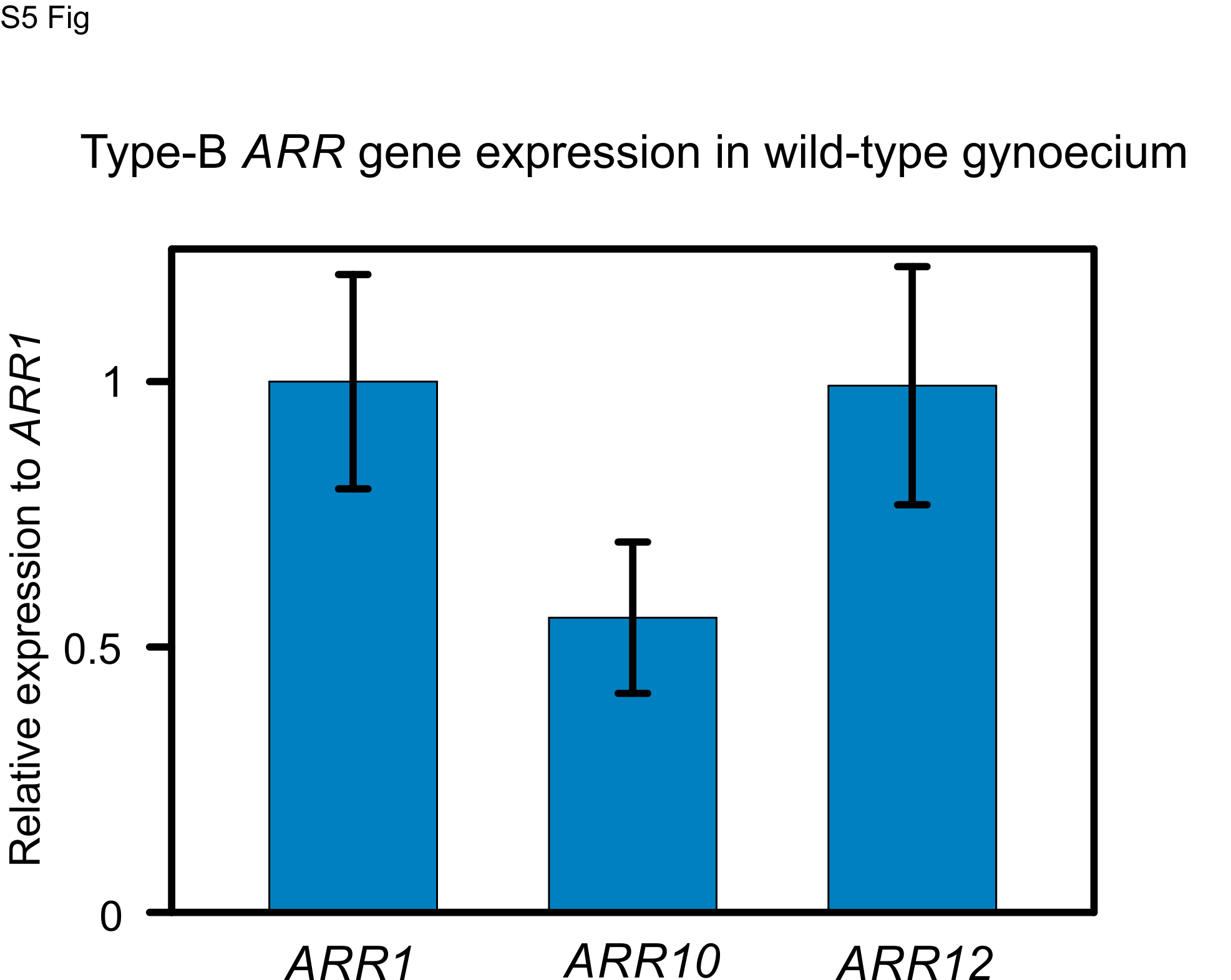

Supplement: S5 Fig — Expression analysis by qRT-PCR of ARR1, ARR10, and ARR12 in wild-type dissected gynoecia. Error bars represent the SD based on three biological replicates. (TIF) [file pgen.1006726.s006.tif]

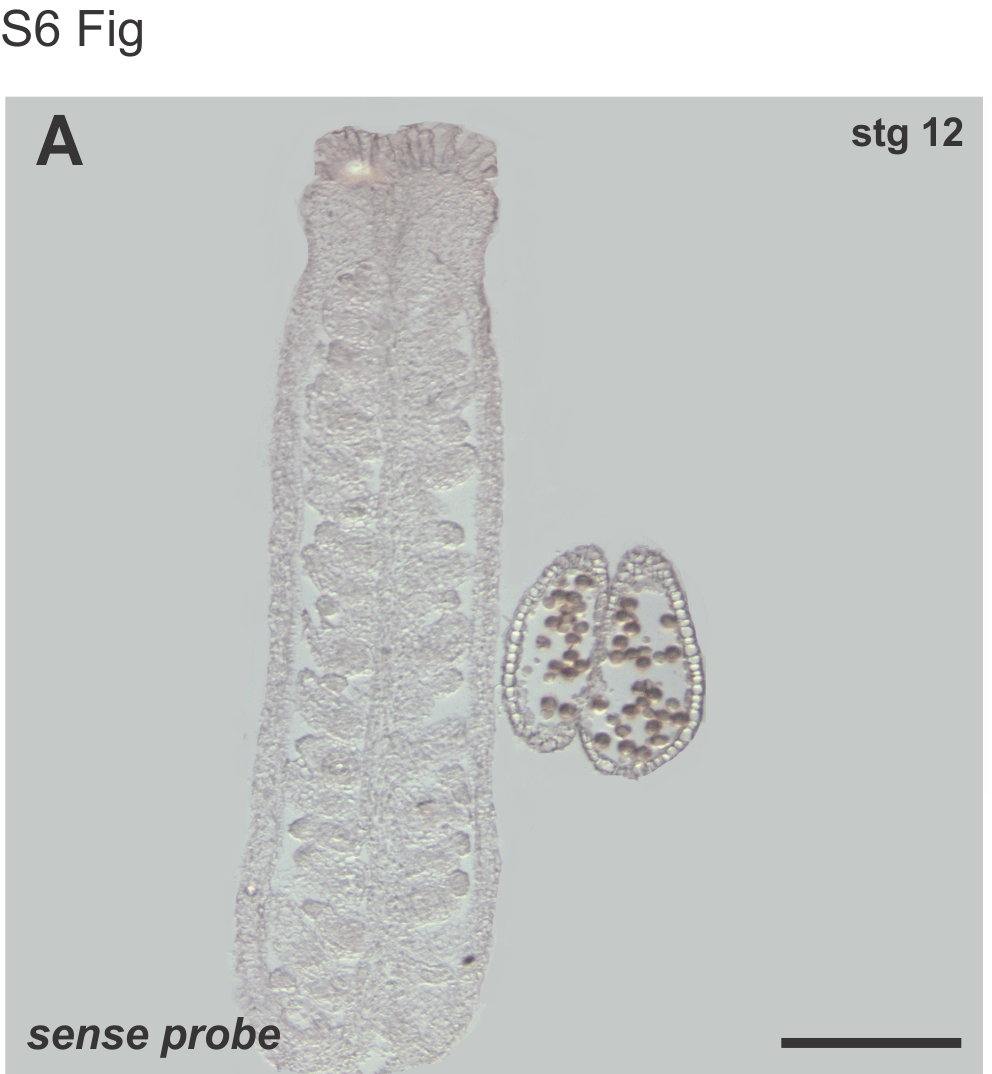

Supplement: S6 Fig — (A) Negative control (sense probe) for the in situ hybridization of the type-B ARR1 in a longitudinal section of a stage 12 gynoecium. Scale bar: 100 μm. (TIF) [file pgen.1006726.s007.tif]

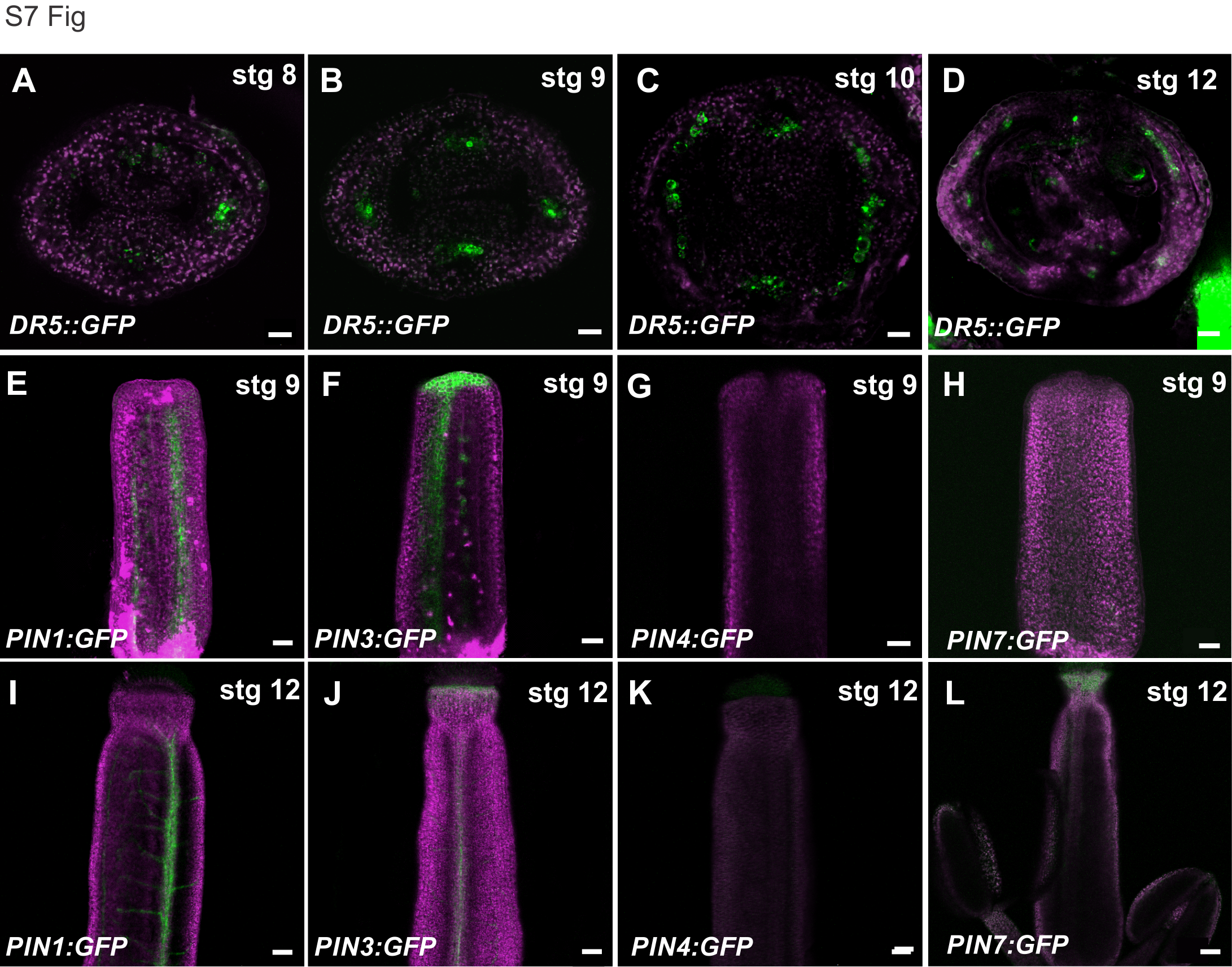

Supplement: S7 Fig — (A-D) Expression of the transcriptional auxin response reporter DR5::GFP line in transverse sections of wild-type gynoecia at stages 8, 9, 10, and 12. (E-L) Expression of PIN translational fusions with GFP in gynoecia at stage 9 and 12: PIN1::PIN1-GFP (E, I), PIN3::PIN3-GFP (F, J), PIN4::PIN4-GFP (G, K), and PIN7::PIN7-GFP (H, L). Scale bars: 10 μm (A-C), 20 μm (D-H), 50 μm (I-L). (TIF) [file pgen.1006726.s008.tif]

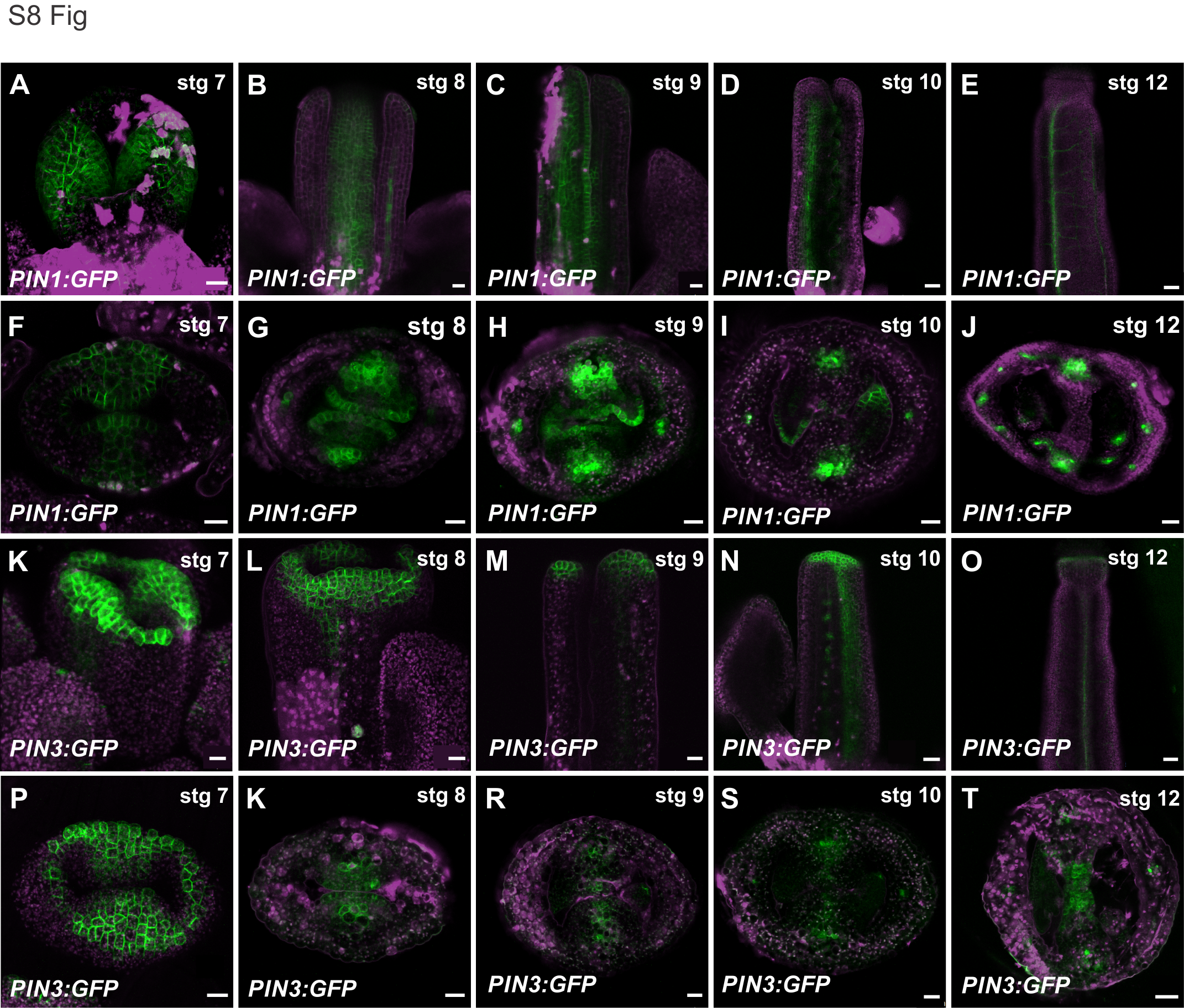

Supplement: S8 Fig — (A-J) The localization of PIN1::PIN1-GFP during gynoecium development at stage 7, 8, 9, 10, and 12 (Longitudinal view: A-E; top view at the apex: F; transverse section in the ovary: G-J). (K-T) The localization of PIN3::PIN3-GFP during gynoecium development at stage 7, 8, 9, 10, and 12 (Longitudinal view: K-O; top view at the apex: P; transverse section in the ovary: K-T). Scale bars: 10 μm (A-C, F-I, K-M, P-S), 20 μm (D, E, J, N, O, T). (TIF) [file pgen.1006726.s009.tif]

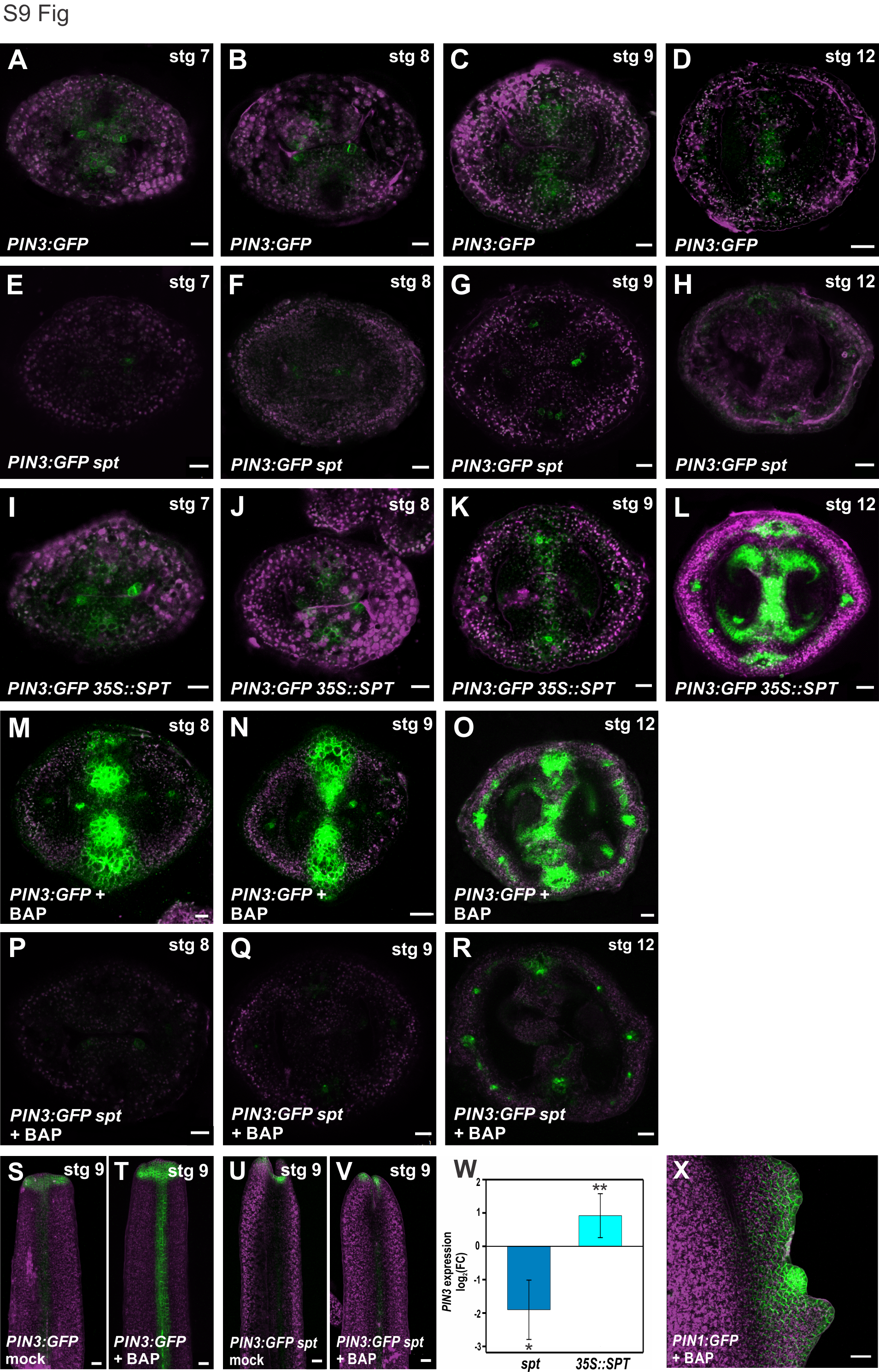

Supplement: S9 Fig — (A-L) Localization of PIN3::PIN3-GFP in transverse sections of gynoecia at stage 7, 8, 9, and 12 of wild-type (A-D), spt-2 (E-H), and 35S::SPT (I-L). (M-R) PIN3 expression after 48 hours BAP treatment of stage 8, 9, and 12 gynoecia in wild-type (M-O) and spt-2 (P-R). (S-V) Longitudinal view of PIN3 expression in a wild-type stage 9 gynoecium (mock) (S) and after 48 hrs BAP treatment (T), and in a spt-2 stage 9 gynoecium (mock) (U) and after 48 hrs BAP treatment (V). (W) Expression analysis by qRT-PCR of PIN3 in dissected gynoecia from spt-12 and 35S::SPT versus wild-type. Error bars represent the SD based on three biological replicates. *P < 0.05, **P = 0.08 (qRT-PCR: ANOVA). (X) Localization of PIN1::PIN1-GFP in the ectopic outgrowths of a gynoecium after five days of BAP treatment. Scale bars: 10 μm (A-C, E-G, I-K, M, N, P, Q), 20 μm (D, H, L, O, R, S-V, X). (TIF) [file pgen.1006726.s010.tif]

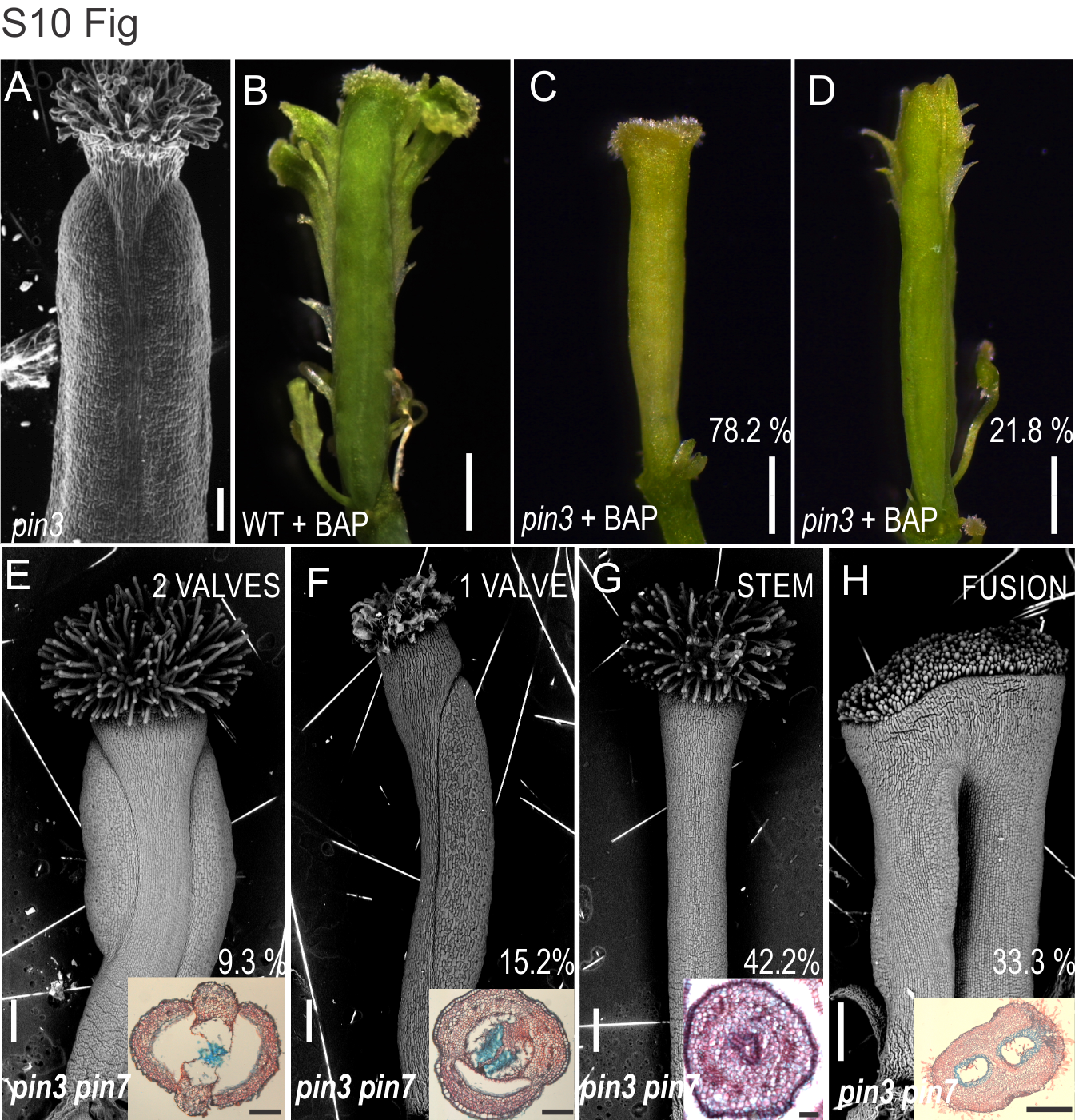

Supplement: S10 Fig — (A) Scanning electron microscopy image of a pin3-4 mutant gynoecium. (B-D) Five days BAP-treated gynoecia phenotypes (photos were taken 3–4 weeks after BAP treatment) of wild-type Col-0 with the typical overgrowth of tissue from the repla (B), pin3-4 lacking the overgrowth of tissue from the repla in 78.2% of the cases (C), and pin3-4 with a slight phenotype in 21.8% of the cases (n = 330) (D). (E-H) Observed gynoecia phenotypes in the pin3 pin7 double mutant (non-treated plants; n = 277). Phenotypes: 9.3% of the cases the size of the carpels is unequal; 15.2% only one carpel present; 42.2% stem-like structure; 33.3% fused gynoecia-like structures. Insets show a transverse section at the middle of the `ovary`structure. Scale bars: 100 μm (A, E-H), 10 mm (B-D). (TIF) [file pgen.1006726.s011.tif]

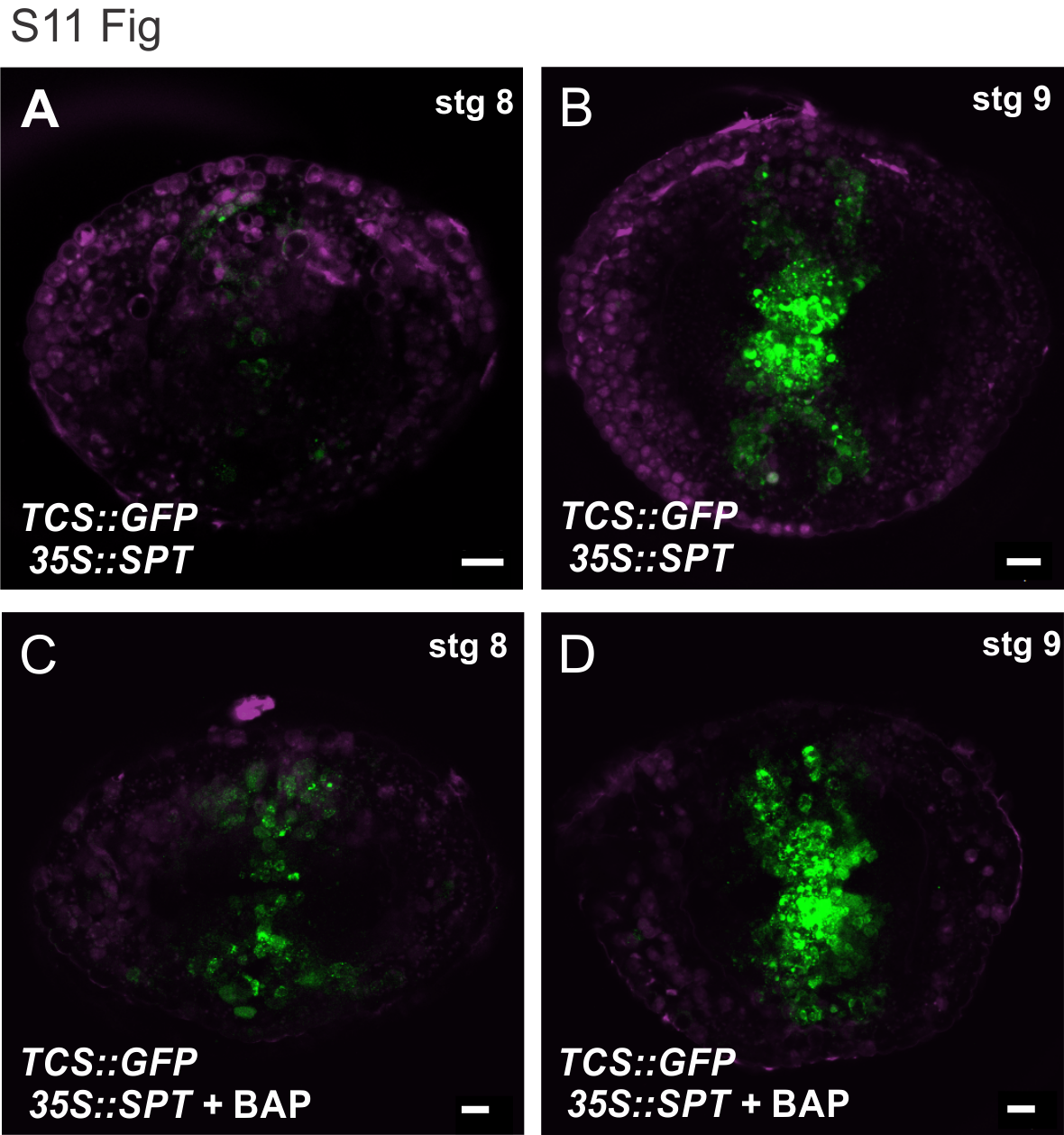

Supplement: S11 Fig — Expression of the cytokinin response reporter TCS::GFP in transverse sections of gynoecia at stage 8 and 9 of 35S::SPT (A, B), and 35S::SPT after 48 hours of BAP treatment (C, D). Scale bars: 10 μm. (TIF) [file pgen.1006726.s012.tif]

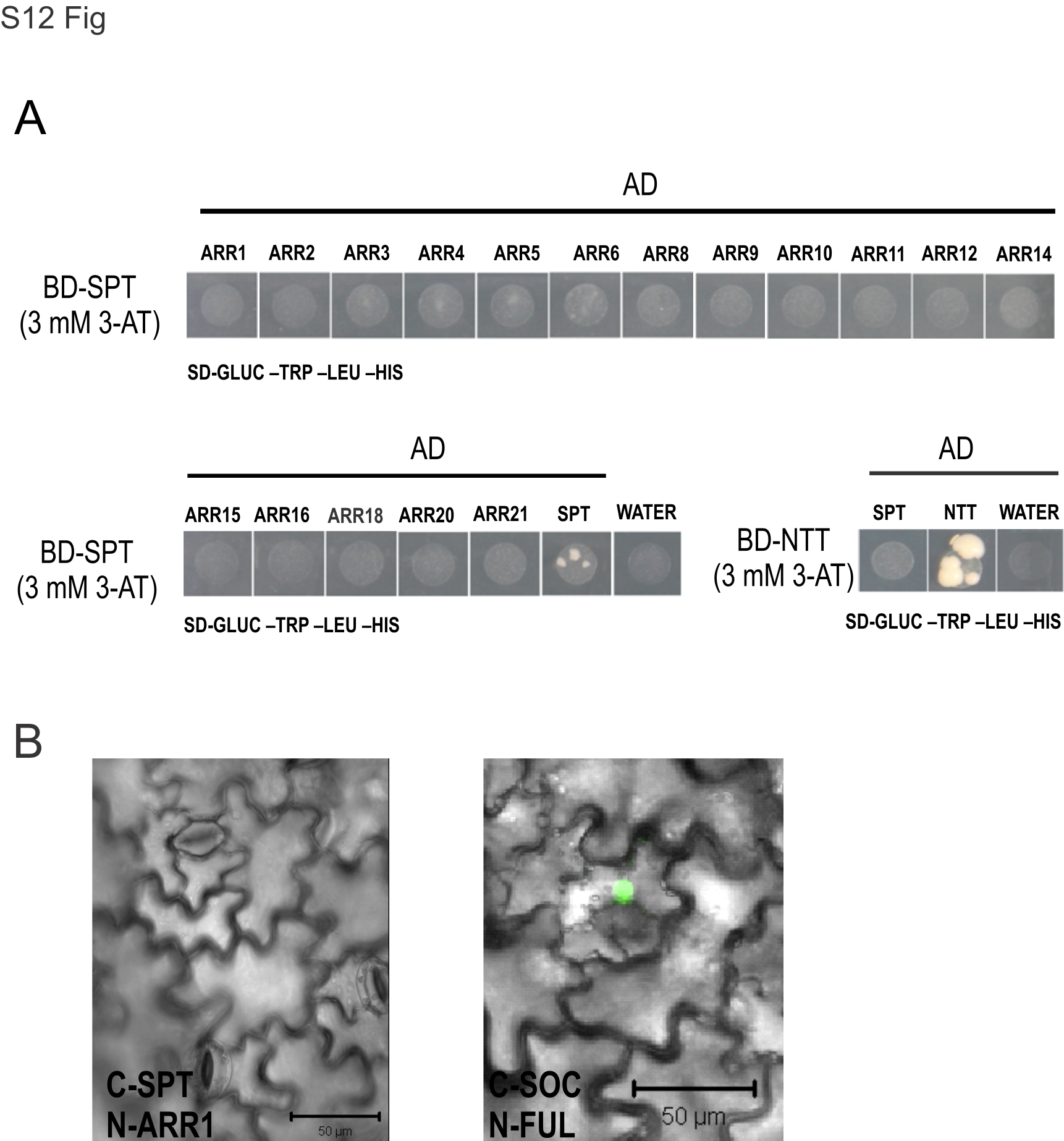

Supplement: S12 Fig — (A) Yeast two-hybrid assay with SPT fused to the GAL4 DNA binding domain in combination with itself (homo-dimerization detection) and with 9 type-B ARR proteins (ARR1, ARR2, ARR10, ARR11, ARR12, ARR14, ARR18, ARR20, and ARR21), and also we performed the assay with 8 type-A ARR proteins (ARR3, ARR4, ARR5, ARR6, ARR8, ARR9, ARR15, and ARR16), all fused to the GAL4 activation domain. Positive control reaction: NO TRANSMITTING TRACT (NTT) fused to the GAL4 DNA binding domain in combination with itself (homo-dimerization detection), and NTT against SPT as a negative control. No interaction is observed between SPT and any tested ARR proteins. (B) Bimolecular fluorescence complementation (BiFC) assay of SPT with ARR1 in N. tabacum leaves, where no interaction (no fluorescence) is detected. Positive control for the BiFC assay is SUPPRESSOR OF OVEREXPRESSION OF CO 1 (SOC1) with FRUITFULL (FUL). (TIF) [file pgen.1006726.s013.tif]
